# Shade Avoidance Components and Pathways in Adult Plants Revealed by Phenotypic Profiling

**DOI:** 10.1371/journal.pgen.1004953

**Published:** 2015-04-15

**Authors:** Kazunari Nozue, An V. Tat, Upendra Kumar Devisetty, Matthew Robinson, Maxwell R. Mumbach, Yasunori Ichihashi, Saradadevi Lekkala, Julin N. Maloof

**Affiliations:** Department of Plant Biology, University of California, Davis, Davis, California, United States of America; University of Minnesota, United States of America

## Abstract

Shade from neighboring plants limits light for photosynthesis; as a consequence, plants have a variety of strategies to avoid canopy shade and compete with their neighbors for light. Collectively the response to foliar shade is called the shade avoidance syndrome (SAS). The SAS includes elongation of a variety of organs, acceleration of flowering time, and additional physiological responses, which are seen throughout the plant life cycle. However, current mechanistic knowledge is mainly limited to shade-induced elongation of seedlings. Here we use phenotypic profiling of seedling, leaf, and flowering time traits to untangle complex SAS networks. We used over-representation analysis (ORA) of shade-responsive genes, combined with previous annotation, to logically select 59 known and candidate novel mutants for phenotyping. Our analysis reveals shared and separate pathways for each shade avoidance response. In particular, auxin pathway components were required for shade avoidance responses in hypocotyl, petiole, and flowering time, whereas jasmonic acid pathway components were only required for petiole and flowering time responses. Our phenotypic profiling allowed discovery of seventeen novel shade avoidance mutants. Our results demonstrate that logical selection of mutants increased success of phenotypic profiling to dissect complex traits and discover novel components.

## Author Summary

Because plants depend on light for photosynthesis, neighboring plant shade can be detrimental to survival. Many plants sense and respond to neighbor shade to compete for light. Although shade causes responses throughout the plant (collectively known as the shade avoidance syndrome or SAS), most SAS studies have been limited to single-gene analyses in seedlings. Here we move beyond these analyses by taking a multi-gene, multi-trait study of SAS across developmental stages. Recently, whole-genome studies examining large mutant collections have been exploited to determine the pathways and their interactions that combine to determine complex phenotypes. This type of analysis (phenotypic profiling) typically uses thousands of mutants and robotic phenotyping for assaying many characters in the multitude of mutant lines. In this paper, we develop a directed alternative that allows us to take a similar approach to understanding SAS. To reduce the number of mutants required for such an approach, we used a logical selection procedure to define mutants of interest by over-representation analysis of shade-responsive genes. We found at least three different subgroups of shade responses, and that each subgroup had both shared and separate pathways. Also, we found eighteen novel genes involved in SAS. Therefore, our method is useful for multi-dimensional phenotypic profiling without expensive robots.

## Introduction

Plant canopy shade limits available light for photosynthesis. Because plants are sessile, this presents a particular challenge. Perhaps as a consequence plants developed a light-quality sensory system for canopy shade; perception of foiliar shade and/or reflection from neighbor plants (“neighbor detection”) can induce the shade avoidance syndrome (SAS) is collection of responses to canopy shade in plants. These SAS responses can be seen in all developmental stages from seeds to adult plants [1]. Various plant organs elongate under shade, including the hypocotyl (stem) of young seedlings, and the internodes, and leaf petioles of older plants. Furthermore shade induces upward leaf movement, accelerates flowering time (the developmental transition form vegetative phase to reproductive phase), suppresses shoot branching, and alters resource allocation [1]. All of these responses can be helpful for promoting survival when there is competition for light from neighboring plants.

Foliar shade, which has reduced photosynthetically active radiation (PAR) can be detected both by cryptochrome photoreceptors due to its reduced intensity of blue light and by phytochrome photoreceptors due to its reduced ratio of red to far-red light [1]. Remarkably, plants can perceive nearby neighbors even before true shading and the concomitant reduction in PAR. This type of neighbor detections is possible because, even though PAR is not reduced, light reflected from neighbors has a reduced ratio of red to far-red light detectable by phytochromes [1,2]. Here we use a neighbor-detection protocol to focus on phytochrome-mediated responses. Detailed analysis of shade induced hypocotyl elongation has revealed that light perception activates transcription factors (TF) that, in turn, modulate plant hormone pathways to promote organ growth. For example, the PHYTOCHROME-INTERACTING FACTOR (PIF) 5 TF protein is stabilized under shade and induces transcription of genes important for synthesis of the growth-promoting hormone auxin [3,4]. Another example is that upon shade treatment PIF7 is dephosphorylated and activated to induce *YUCCA* (*YUC*) *2*, *YUC5*, *YUC8*, and *YUC9* auxin biosynthetic genes [5].

Plant hormones regulate many aspects of development and growth. At least five plant hormone pathways are related to SAS; auxin, brassinosteroid (BR), gibberellic acid (GA), ethylene, and cytokinin (CK). Many auxin or BR responsive genes are induced by end-of-day far-red treatment (EODFR, a proxy for shade treatment) and both auxin (*big* and *shade avoidance 3* (*sav3*) */ tryptophan aminotransferase of Arabidopsis 1* (*taa1*)) and BR (*rotundifolia3* (*rot3*)) mutant showed reduced shade-induced or EODFR-induced petiole elongation as well as shade-induced gene expression [6,7]. There is some evidence that GA and CK are involved in leaf SAS [8,9,10]. Shade also influences jasmonic-acid (JA) mediated plant immune system [11] and reduced volatile JA levels [12]. However, the entire network of light signaling and hormone pathways in regulation of shade avoidance are unclear. Also, the extent of shared and separate pathways for each shade avoidance response is not currently known. In part this is because each SAS mutant has been tested under different experimental conditions making it difficult to compare the phenotypic consequences of each mutant.

Phenotypic profiling of genetic mutants with high-throughput phenotyping is a powerful method to tease out complex gene networks [13]. Systematic phenotypic profiling of multiple traits originated with bacterial studies, followed by studies on single eukaryotic cells such as yeast and cultured animal cells [14]. Multi-dimensional phenotypic profiling of gene-perturbed multi-cellular organisms had been done both in invertebrates and vertebrates [15,16,17]. In plants phenotypic profiling of recombinant inbred lines or natural population has been conducted for QTL analysis or genome-wide association studies [18], but it has not yet been applied to induced mutants in plants.

Advances of automatic and robotic technologies made it possible to conduct high-throughput phenotyping. In plant, high-throughput robotic phenotyping systems had been reported (reviewed in [19,20]), but its use in research has only recently been published [21]. Profiling of multiple phenotypes in selected gene-perturbed plants has been reported in root epidermal cell patterning study [22] and red-light signaling [23,24], which showed effectiveness of reverse-genetics approaches to recover mutants of interest from transcriptome data.

Here we extend this approach to multiple phenotypes to develop a systems-level understanding of shade avoidance. We took advantage of a newly developed semi-automated leaf shape measurement system for throughput measurement of shade avoidance in leaves [25]. Furthermore, we aimed to narrow down candidate genes involved in SAS to be screened instead of screening of entire knockout mutant collection or mutagenized population. For this purpose, we selected candidate mutants based on over-representation analysis of shade-responsive genes in leaves. Our broad phenotypic profiling of hypocotyl, leaf, and flowering time in selected mutants has allowed us to dissect the complex shade avoidance syndrome network.

## Results and Discussion

### Shared and separate gene sets are enriched in shade-responsive genes in *Arabidopsis* seedling and leaf/apical region

To begin to define the genes required for SAS in leaf/apical region, after the seedling stage we performed an expression profiling experiment to find gene induces or repressed by simulated shade. Previous SAS expression profiling experiments have used microarrays and focused on seedlings or specifically on the petiole or leaf blade after EODFR treatment [6,7,26]. To obtain a broader view of expression changes in older plants, we harvested leaf and shoot apex tissue and used RNAseq (statistics are presented in S1 Table) since the most common *Arabidopsis* microarray (Affymetrix ATH1) only covers about 70% of defined genes in the transcriptome. We compared gene expression in samples treated with simulated shade (white light supplemented with far-red light to achieve R/FR of 0.5 and 80–100 μE PAR) for 1 hour or 4 hours to untreated control samples (R/FR = 1.9 and 80–100 μE PAR) and found a total of 164 and 97 genes to be differentially expressed (FDR <0.001; S2 Table see Materials and Methods). Most known shade induced genes in leaves were found in our list (bold text in S2 Table, 1 hour after onset of shade treatment) [3,4,7,27,28,29]. There is a high correlation of expression fold changes by shade treatment between our data and published microarray data in leaf [6] (S1 Fig), confirming that our RNA-seq based transcriptional analysis is reliable. Significant correlation of shade-responsive genes between seedlings and leaves indicates common mechanisms exist between these two organs (S1 Fig). In addition we identified 38 (1 hour treatment) and 19 (4 hour treatment) genes not present on the *Arabidopsis* ATH1 microarray, including the known shade induced gene *PHYTOCHROME-INTERACTING-FACTOR-3 LIKE I* (*PIL1*) [3,30], while 50 (1 hour) and 68 (4 hour) genes were on ATH1 but not previously found as shade-responsive genes in EODFR treated petiole or leaf (S2 Table).

GO enrichment analysis showed that plants respond to 1 hour and 4 hour shade treatment differently (Tables 1 and 2). Two GO terms common to both time points were GO:0009733 (response to auxin stimulus) and GO:0009753 (response to JA stimulus). Known shade avoidance related genes were also enriched in the 1 hour treatment, but not in the 4 hour treatment. Plant immune related pathways (GO:0009611 (response to wounding) and GO:0009617 (response to bacterium), which are related to the JA pathway, are enriched in the 4 hour treatment.

**Table 1:** GO category analysis of 1 hour shade-responsive genes in leaf/apical region.

category	Term	over_represented_pvalue	over_represented_padjust value
GO:0009733	response to auxin stimulus	2.70e-41	6.70e-38
GO:0010583	response to cyclopentenone	7.49e-07	6.61e-04
GO:0009753	response to jasmonic acid stimulus	8.00e-07	6.61e-04
GO:0009641	shade avoidance	3.03e-06	1.88e-03
GO:0009630	gravitropism	5.48e-06	2.72e-03
GO:0009741	response to brassinosteroid stimulus	7.92e-06	3.27e-03

Plants were grown on soil under simulated sun (R/FR = 1.9) and long-day (16/8 hours) conditions for two weeks and then were transferred to simulated shade (R/FR = 0.5) or left in the sun condition. Above-ground parts excluding hypocotyl were collected at 1 hour shade treatment in leaf. Terms with adjusted p-value by Benjamini & Hochberg method [135] <0.01 were selected from GOseq analysis.

**Table 2:** GO category analysis of 4 hour shade-responsive genes in leaf/apical region.

category	Term	over_represented_pvalue	over_represented_padjust value
GO:0009753	response to jasmonic acid stimulus	5.27e-10	1.31e-06
GO:0009611	response to wounding	2.29e-09	2.84e-06
GO:0055114	oxidation-reduction process	2.75e-07	2.27e-04
GO:0080167	response to karrikin	2.08e-06	1.10e-03
GO:0009733	response to auxin stimulus	2.21e-06	1.10e-03
GO:0009617	response to bacterium	2.92e-06	1.21e-03

Same as Table 1 except 4 hour shade treatment instead of 1 hour shade treatment

It has been described that some plant hormone pathways, such as auxin, BR, and GA, are involved in SAS [1]. To gain a better understanding of involvement of hormone pathways in SAS, over-representation analysis (ORA) was done to test if any hormone-responsive genes were enriched among the shade-regulated genes (Table 3). Consistent with ORA with previous microarray data of leaf upon EODFR treatment, auxin, BR, and JA pathways were enriched [6]. In addition, we found that ethylene and abscisic acid (ABA) pathways were enriched in our data sets. Involvement of ethylene in SAS was suggested because shade increases ambient ethylene levels in *Sorghum* [31] and tobacco [32]. However ethylene production is required only for early response to shade in petiole and stem, but not response in leaf angle [32], although ethylene induces leaf hyponasty [33], hypocotyl elongation [34], and stem elongation [35]. Involvement of ABA in SAS is known for shade-suppressed branching [36], but has not been reported to be involved in the shade-avoidance responses examined in this paper. Interestingly leaves of four-day shade treated tomato plants have increase level of ethylene precursor and ABA [37]. In summary, the significant differences in the shade-responsive transcriptome at these two time-points reflected dynamic temporal changes of early SAS signaling cascade.

**Table 3:** Over-representation analysis of shade-responsive genes and hormone responsive genes.

	1 hour shade treatment		4 hour shade treatment	
regulated by hormone	UP	DOWN	UP	DOWN
**IAA UP**	4.59e-75	1	1.2e-06	0.138
**IAA DOWN**	1	0.0642	0.152	1
**BR UP**	0.000269	1	1	1
**BR DOWN**	1.04e-10	1	0.00355	1
**GA UP**	1	1	1	1
**GA DOWN**	0.00148	1	1	1
**JA UP**	0.0083	0	8.34e-18	0.0567
**JA DOWN**	1.9e-12	1	0.000406	1
**CK UP**	0.00259	1	1	1
**CK DOWN**	1	1	1	1
**ABA UP**	0.0258	0.431	1.31e-07	1
**ABA DOWN**	0.00141	0.252	0.0064	1
**ACC UP**	1.58e-05	1	0.000106	1

p-values were calculated by GOseq [129] and adjusted by Benjamini & Hochberg correction [135].

Next we asked if the previous seedling shade transcriptome data [7] shows the same trends as our data. We found that there were both common and specific GO terms between the hypocotyl and our leaf/apical region data sets (Table 1, 2, and 4). Of the common terms, we focused on GO:0009733 (response to auxin stimulus), GO:0009741 (response to BR stimulus), and GO:0009641 (shade avoidance). Among the leaf/apical region-specific terms GO:0009753 (response to JA stimulus) is of particular interest because the role of JA pathways in morphological aspects of SAS is not fully understood. Based on our ORA we chose to include mutants of genes in these categories in our phenotypic profiling (see below).

**Table 4:** GO category analysis of 1 hour shade-responsive genes in hypocotyl.

category	Term	over_represented_pvalue
GO:0009719	response to endogenous stimulus	1.72e-38
GO:0010033	response to organic substance	1.46e-32
GO:0009725	response to hormone stimulus	9.18e-32
GO:0009733	response to auxin stimulus	6.02e-28
GO:0042221	response to chemical stimulus	3.04e-24
GO:0050896	response to stimulus	1.23e-17
GO:0009741	response to brassinosteroid stimulus	5.38e-16
GO:1901700	response to oxygen-containing compound	2.03e-12
GO:0033993	response to lipid	7.38e-10
GO:0097305	response to alcohol	1.25e-09
GO:0009639	response to red or far red light	2.94e-09
GO:0014070	response to organic cyclic compound	4.45e-09
GO:0065007	biological regulation	1.62e-08
GO:0009612	response to mechanical stimulus	2.55e-08
GO:0009641	shade avoidance	5.70e-08
GO:0010200	response to chitin	2.78e-07
GO:0010243	response to organic nitrogen	4.77e-07
GO:0009416	response to light stimulus	8.17e-07
GO:0009314	response to radiation	2.87e-06
GO:0009628	response to abiotic stimulus	4.51e-06

Same as Table 1 except 1 hour shade treatment in hypocotyl instead of 1 hour shade treatment in leaf. Expression data is from [7]. Terms with p-value<1e-5 were selected from amiGO analysis.

### Leaf phenotype profiling of fifty-nine mutant lines

**Fig 1:**
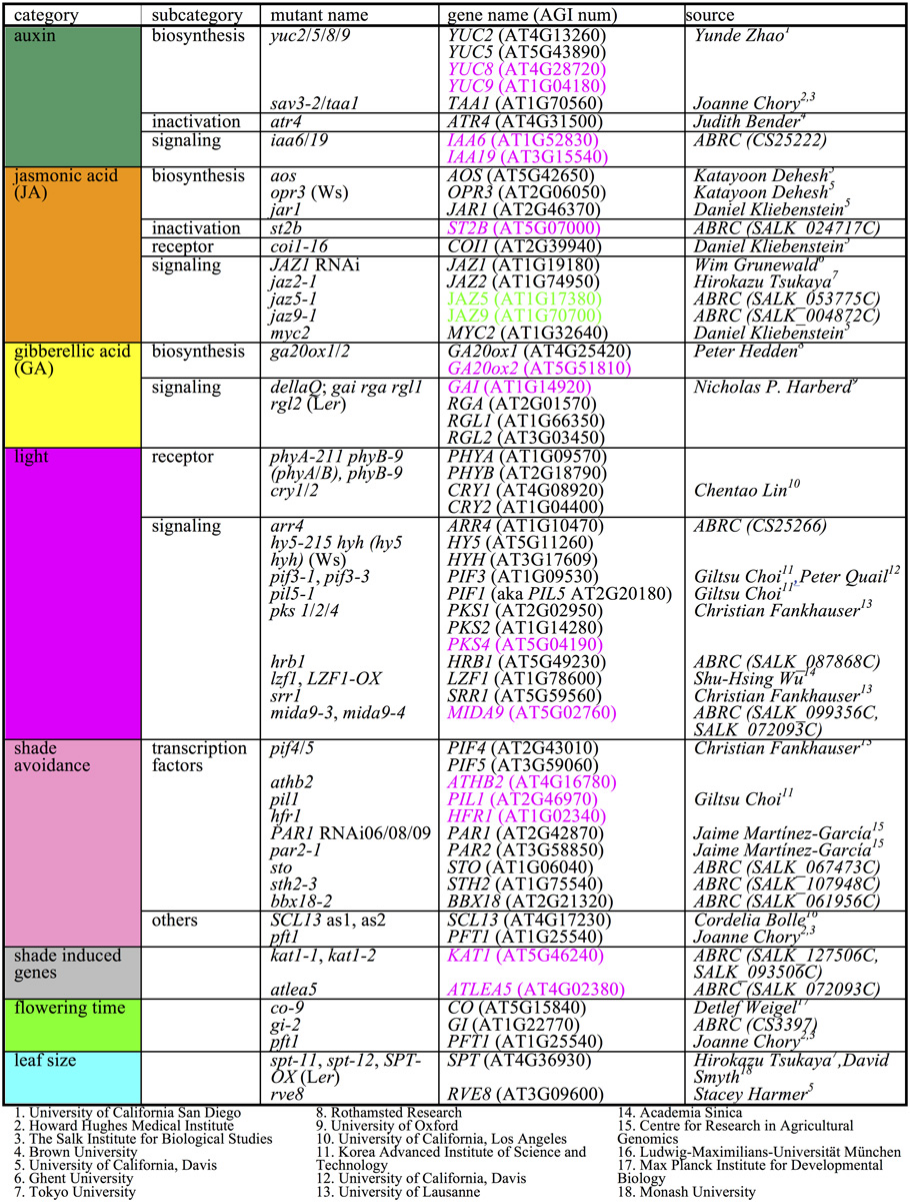
Mutants used in SAS phenotypic profiling. Shade-induced genes are shown in magenta bold and shade-repressed are shown in green. Background genotype for almost all mutants is Col (other genotypes are show in parentheses).

We hypothesized that shade-responsive genes and/or pathways are required for proper SAS because among the 34 causal genes in known hypocotyl SAS mutants 11 (*TAA1*, *PIN3*, *YUC2*, *YUC5*, *YUC8*, *YUC9*, *BIM1*, *GAI*, *PHYB*, *PAR1*, *HAT3*) of them have shade-responsive transcripts (S3 Table). Based on our differentially expressed gene list and previous knowledge, we chose 59 mutant lines encompassing 59 mutant genes (although some lines have more than one mutant gene) from nine categories (auxin, GA, JA, BR, light signaling, shade avoidance, flowering time, leaf size, and unknown shade responsive genes) (Fig 1). We prioritized auxin and JA pathways because both pathways were enriched in both time points of our transcriptome analysis (see above).

Petiole elongation is an important component of SAS, but most leaf phenotype measurement software does not report petiole and blade length. Phenotyping of mutants/overexpressors with a pathway of interest is a direct method to test if the given gene in the pathway is involved in phenotype of your interest. At the first step towards high throughput petiole and blade phenotyping, we developed LeafJ, an ImageJ plug-in, which is more accurate and faster measurement system than manual method [25].

**Fig 2:**
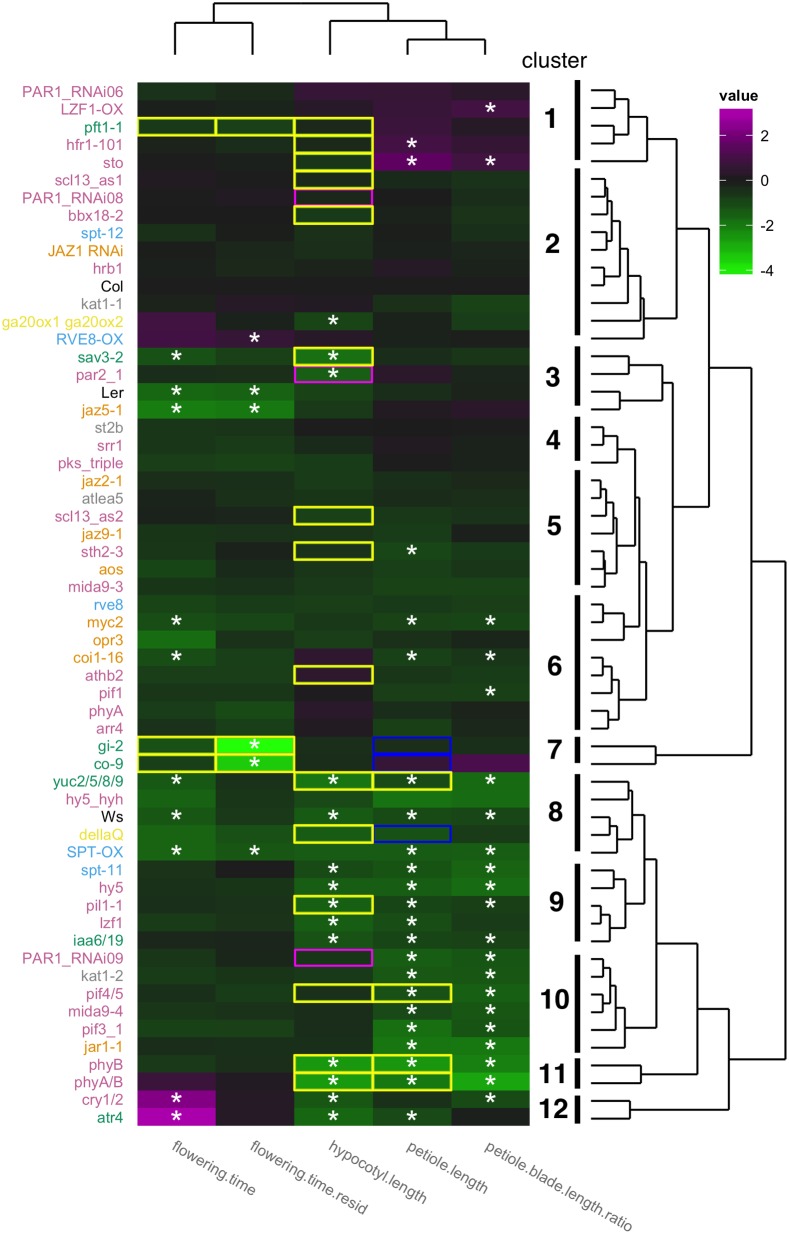
Phenotypic profiling of 59 mutants/overexpressors. For hypocotyl phenotype, plants were grown under continuous simulated sun conditions (R/FR = 1.3) for four days and further grown for three days under either simulated sun or simulated shade (R/FR = 0.5). For leaf phenotypes, plants were grown under long day conditions (16 hour light/8 hour dark) with approximately 90 μE PAR (R/FR = 1.9). Two week old plants were further grown for 12 days under either simulated sun (R/FR = 1.9) or simulated shade (R/FR = 0.5). For flowering time phenotype plants were grown in the same condition with leaf phenotyping and days after stratification at bolted was used for flowering time index. For phenotype clustering heatmap, differences between shade and sun values (except flowering time) were normalized and centered on Col (i.e., Col value = 0) and visualized with color coding (magenta indicates larger response than Col while green indicates reduced response relative to Col). For flowering time, responses to shade were normalized against flowering time under sun condition to eliminate strong dependencies of response on flowering time under sun condition (see S2H Fig for normalized data and S2G Fig for non normalized data). Asterisks (*) from hypocotyl.length to flowering.time indicate a significant difference from corresponding wild type (p-value<0.05). Although SPT_ox appeared to be a SAS mutant in flowering time (residual method, S3H Fig), it was eliminated from SAS mutants in flowering time (residual method) because its background genotype (L*er*) also showed a similar shift from the regression line. Colors of mutant names correspond to groups found in Fig 1. Clustering of mutants according to its phenotype is shown in the dendgrogram on the right. Clustering of traits is show on the top dendrogram. Known phenotypes for hypocotyl, petiole, or flowering time are shown in colored boxes (yellow-green for less response, blue for normal response, and magenta for exaggerated response).

To normalize petiole elongation between genotypes with different leaf size, we calculated ratio of petiole length to blade length. Statistical significance between Col under sun treatment and shade treatment effects was examined by a mixed effects model (Fig 2). Mutants that showed a statistically different response to shade when compared to the corresponding wild-type (P<0.05; see Methods) were considered to have a significant SAS phenotype. In Col, we could detect significant shade-induced petiole elongation and an increase of the petiole length to leaf blade length ratio (S2F Fig and S3 Table), but not in other leaf blade parameters (length, width, and area) (S2C–S2E Fig). Some other studies have reported that leaf area does increase or decrease upon shade treatment; the differences between these studies and ours may be due to differences in plant growth conditions or because the developmental stage of our leaves might be too young to show these responses [7,38,39,40]. Comparing the kinetics of leaf development under both light condition could tell us when shade-responsive organ elongation happens and should be examined in future studies.

Screening these 59 mutant lines, we found 33 mutants that showed differences in at least one trait from the background ecotype (Fig 2, and S3 Table). These 33 mutants include genes in the auxin, jasmonic acid, and light signaling pathways. Involvement of auxin in petiole elongation upon far-red light treatment has been previously reported [5], whereas there have not been any reports of JA involvement in petiole shade avoidance. Details of these mutants will be discussed below. It is important to note that we assay a leaf series from each plant where some leaves are still expanding and others are mature. As a consequence, the mutations that we have identified could be affecting leaf development itself or developmental timing (the proportion of expanding to expanded leaves).

### Shade-induced acceleration of flowering time of fifty-nine mutant lines

Compared to other SAS phenotypes, only seven mutant lines for acceleration of flowering time have been described; phytochrome mutants (*phytochromeB* (*phyB*) *phyD*, *phyB*/*D*/*E)* [41,42], known flowering time mutants (*constans* (*co*) and *gigantea* (*gi*)) [43], a circadian clock component (*early flowering 3* (*elf3*)) [44], an auxin-related mutant (*big1*) [45], and a mediator complex mutant (*phytochrome and flowering time 1* (*pft1*)) [46]. Our SAS phenotypic profiling provided additional mutants for shade-accelerated flowering. In our condition shade treatment accelerated flowering time about 10% in Col, which is less effective than previously reported (35–40% [42,43,47]). This small acceleration is because our shade treatment started when plants were already close to flowering in long day conditions. Perhaps because of this we observed a strong inverse correlation between flowering time shade response and flowering time in sun (S3H Fig; genotypes that flowered later in sun were more responsive to shade). Given this correlation we defined two categories flowering time shade response mutants. One category (“flowering.time” in Fig 2) consists of mutants whose log2 shade response is different from Col-0. The second category (“flowering.time.resid” in Fig 2) are those mutants whose response is significantly different from that expected based on their flowering time in the sun (see Methods). Even though our experimental conditions were suboptimal for detecting acceleration of flowering time and we could not reproduce reduced responses to shade of *pft1*, we did find six new flowering time SAS mutants; *cryptochrome* (*cry*) *1 cry2 (cry1*/*2)*, *altered-tryptophan regulation 4* (*atr4)*, *reveille 8*/*lhy-cca1-like 5* (*rve8*/*lcl5*), *coronatine insensitive 1* (*coi1)*, *myc2*/*jasmonate insensitive1* (*jin1*), and *jasmonate-zim-domain protein 5* (*jaz5)* (Fig 2, and S3 Table). Details of these mutants will be discussed below.

### Separate and overlapping pathways for three different shade avoidance responses

To investigate how many of the genes under study are required for normal shade avoidance response in both the leaf and the hypocotyl, we assayed hypocotyl SAS in the same 59 mutant panel (S3A Fig, Fig 1, and S3 Table). Among the eighteen previously reported hypocotyl SAS mutants tested, we observed altered SAS phenotypes in six mutants (28%). This relatively low validation rate is probably due to differences in growth conditions such as day length, the ratio of red to far-red used for sun and shade, or whether shade was applied throughout the day or simulated by EODFR. In addition to previously described SAS mutants, we discovered seven mutant lines with previously unknown hypocotyl SAS defects (*gibberellin 20-oxidase* (*ga20ox*) *1 ga20ox2* (*ga20ox1*/*2*), *phytochrome rapidly regulated* (*par*) *2–1*, *indole-3-acetic acid inducible* (*iaa*) 6 *iaa19 (iaa6*/*19)*, *light-regulated zinc finger protein 1* (*lzf1*), *spatula* (*spt*), *elongated hypocotyl 5* (*hy5*), *cry1*/*2*, and *atr4*).

Our mutant phenotypic profiling also revealed that two different indices for one phenotype (“petiole length” and ratio of “petiole blade length ratio” for SAS in petiole; “flowering.time” and “flowering.time.resid” for SAS in flowering time) were clustered together, two elongation phenotypes (hypocotyl elongation and petiole elongation) were clustered while the two elongation phenotypes and flowering time were distinct (top dendrogram in Fig 2). Examining which genes have mutant phenotypes for each trait revealed that there are common and separate pathways for each shade avoidance response (Fig 2 and S3 Table). Auxin-related genes are required for all responses; phytochromes and three TFs (*LZF1*, *HY5*, and *PIL1*) were involved in both hypocotyl and leaf responses; cryptochromes are involved in both hypocotyl and flowering-time response; and JA related pathways were involved in petiole and flowering time responses. Twelve genes were required specifically for petiole response and two genes were required specifically for hypocotyl response (Fig 2 and S2 Table). Our results also showed that our strategy was powerful for identifying new genes required for these shade avoidance responses, including genes in light, auxin, JA, and BR pathways (S2 Table). Details of those genes will be discussed in following sections.

Differences in the sets of genes required for shade avoidance response in hypocotyls and leafs are consistent with GO analysis of shade-responsive genes in hypocotyl or leaf tissue (Table 1, 2, and 4). For example GO:0009753 (response to JA stimulus) is only found in leaf data sets and we found that mutants affecting the JA pathway only affected SAS phenotypes in adult plants. There is a previous report of JA affecting seedling SAS, but this was under extremely low R:FR (0.068) and JA was found to act by modulating phyA signaling [48]. Under the more moderate low R:FR conditions used in this study and for the seedling microarray assays [7], phyA is not involved (Fig 2) and phyB is the major receptor for shade. Thus, under moderate shade conditions, JA is likely to affect adult rather than seedling SAS.

Our findings that pathways of flowering time in response to shade were different from those of hypocotyl are consistent with previous data. For example, altered *ARABIDPSIS THALIANA HOMEOBOX PROTEIN 2* expression changed shade-avoidance responses in hypocotyl [49] but not in flowering time [43]. In addition, *supressor of phytochrome a-105* (*spa1*/*2*/*3*/*4*) quadruple mutant and *constitutive photomorphogenesis 1* (*cop1*) show shade-induced acceleration of flowering time, but did not show shade-induced hypocotyl elongation [50].

### Updated shade avoidance syndrome pathways

**Fig 3:**
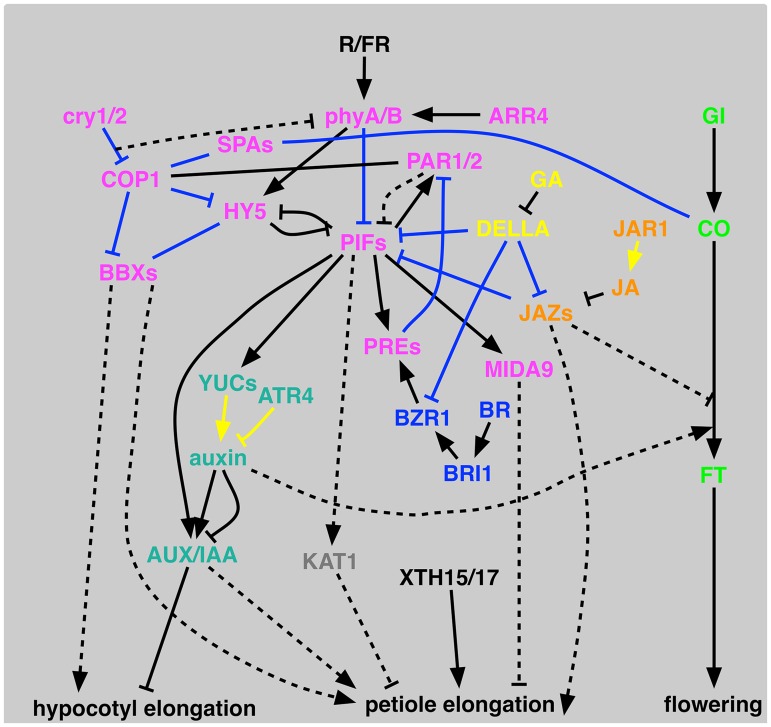
Schematic representation of proposed signal transduction for shade-avoidance syndrome. PIFs represent PIF3, PIF4, PIF5, PIF7, PIL1, and SPT. JAZs represents JAZ5 and JAZ10. Black lines represent genetic interactions, blue lines show direct interactions, and yellow lines show hormone biosynthesis/metabolism. Lines without arrowheads are for interactions where directionality is unknown. Dashed lines show hypothetical interactions. SCL13 is omitted because of its unknown function within this context.

Among 59 tested mutant lines we could detect 33 mutants with defects in at least one shade avoidance response, including 20 new mutant lines (Fig 2). A schematic diagram of SAS signaling pathways is shown in Fig 3. SAS phenotypes with mutants used in this study and known SAS mutants are summarized in S3 Table. Below we discuss details of the pathways corresponding to each mutant category in Fig 1 and then discuss phenotypic clustering of SAS mutants (Fig 2).

#### Light signaling pathway

We simulated canopy shade solely by changing the R/FR ratio, which is perceived by phytochromes. Five *PHY* genes in *Arabidopsis thaliana* have partially overlapping functions [51]. Our phenotypic profiling showed *PHYB* is the major photoreceptor and *PHYA* has minor function with *PHYB* in the SAS responses of hypocotyl and leaf in low R/FR condition because *phyA* mutant phenotypes could be observed only in a *phyB* mutant background, consistent with previous data [1] (S3 Table, S3A, S3B, and S3F Fig). The situation is different for flowering time where *PHYB* does not dominate the response, instead it is redundant with *PHYD* and *PHYE* [47]. This is consistent with our data that *phyB* and even *phyA*/*B* showed normal shade acceleration of flowering time (Fig 2).

We found that the CRY blue-light photoreceptors were important for proper hypocotyl and flowering time SAS under simulated canopy shade. At first this is surprising because our simulated sun and shade conditions only have altered R/FR ratios but have the same blue irradiance. However, CRY1 and CRY2 blue light photoreceptors of *Arabidopsis thaliana* are known to interact genetically with phytochrome signaling in other photomorphogenic responses [52,53,54,55,56,57,58,59,60]. There are numerous molecular events known to occur upon phytochrome activation that may explain functional interaction between phytochromes and cryptochromes. One example is light-dependent PHYB-CRY1 interaction [61] and light dependent CRY-COP1 E3 ligase interaction [62]. Recent studies showed COP1 and its interacting proteins SPA1/2/3/4 are also required for SAS in hypocotyl [50,63,64], so that COP1-SPAs system could be a key component of phytochrome-cryptochrome interaction observed in our data. Although the *cry1*/*2* double showed reduced SAS in hypocotyl and reduced acceleration of flowering time, the mode of action for these two traits are different. *cry1*/*2* hypocotyls showed constitutive shade avoidance, while *cry1*/*2* showed constitutive exaggerated “sun” phenotype for flowering time, indicating that the interaction between phytochrome and cryptochrome signaling are different between these two traits. Possible mechanisms of the differences could be related to photoperiod-dependent flowering time regulation by CRY2 [65]. CRY2 interacts with SPA in blue-light dependent manner, which prevents CO degradation by COP1-SPAs [66]. Also CRY activates a subset of bHLH TFs in a blue light dependent manner; these bHLHs in turn activate the flowering time master gene, FT [67]. Since cryptochromes are required to sense depleted blue light under canopy shade [68,69], plants may have evolved a cross-talk system between phytochrome and cryptochrome signaling systems to coordinate shade avoidance syndromes response in natural condition.

Among TFs in light signaling pathways that we tested (Fig 1), our data showed that *HY5*, *LZF1*, *PIL1*, and *SPT* were required in common between hypocotyl and petiole responses, while *PIF1*, *PIF3*, *PIF4*, *PIF5*, *PAR1*, *LONG HYPOCOTYL IN FAR-RED (HFR1)*, *SALT TOLERANCE* (*STO*), and *SALT TOLERANCE HOMOLOG2*/*B-BOX DOMAIN PROTEIN 21 (STH2*/*BBX21)* were required in petiole response and *PAR2* was required in hypocotyl.

Among them *SPT* is of particular interest because of its unique spatial expression pattern at the boundary between the leaf blade and petiole, where petiole cell proliferation occurs [70]. Thus the spatial expression pattern of *SPT* could explain the reduced SAS petiole elongation phenotype of *spt-11*. Also the severe allele of *SPT* mutant (*spt-11*) showed less shade avoidance responses than mild allele (*spt-12*) (S3 Table), consistent with the reported differences of petiole length and leaf blade area phenotype between *spt-11* and *spt-12* [70]. *SPT* regulates gynoecium development by activating genes involved in shade avoidance [71]. Similar mechanisms of *SPT* regulation of shade avoidance are likely conserved in shade-induced petiole elongation.

It is well recognized that PIFs are growth-promoting proteins whose activities are modulated by a variety of environmental factors [72]. In addition to *PIF4* and *PIF5*, our data showed that both *PIF1* and *PIF3* are new positive regulators of SAS in petiole (Fig 2). *PIF1* is known to regulate hypocotyl growth [72,73] as well as non-growth related processes such as chlorophyll biosynthesis [74]. *PIF3* is also known to regulate hypocotyl growth (reviewed in [75]), but the role of either gene in regulating adult plant growth is not known. We confirmed the *pif3* result by examining a second allele, *pif3-3* [76], and found that *pif3-3* also had a significant petiole SAS defect (S4 Fig; p<0.01). In hypocotyl growth, PIF1/3/4/5 regulate overlapping and specific target genes [77], and it seems likely that many of these targets also contribute to petiole SAS.

We found that a knock-out mutant of a PP2C-type phosphatase gene, *MISREGULATED IN DARK* (*MIDA) 9*, showed reduced petiole shade response. *MIDA9* has been shown to be involved in hook formation and its expression is regulated by *PIF3* [23], consistent with our finding that *pif3* mutant also showed reduced petiole shade response. We previously found that *MIDA9* is induced by *PIF4* and/or *PIF5* during hypocotyl growing phase [78], suggesting that *MIDA9* is involved in growth-control networks. Another link is inactivation of H^+^-ATPase activity (required for cell wall loosening) via dephosphorylation of H^+^-ATPase by MIDA9 phosphatase 2C-typeD [79]. It would be interesting to know if other *PIFs* and *SPT* controls *MIDA9* expression.

#### Auxin pathways

Previous studies have implicated auxin as being important in hypocotyl and leaf SAS [80]. Here we extend those studies to identify specific auxin signaling components important for SAS in various organs. Consistent with prior studies [4,5,6,7], both our transcriptome data and mutant phenotypic profiling showed auxin pathways were involved in hypocotyl and leaf shade avoidance responses. By assaying many auxin pathway genes we can begin to assign shade avoidance functions to specific components.

Our data showed that components of auxin pathway required for each response are different. For example, two different gene families in one auxin biosynthesis pathway contribute differently in three separate shade avoidance responses. Although TAA1 protein catalyzes auxin biosynthesis in the same pathway as YUC proteins [81,82], *TAA1* was required only for hypocotyl shade avoidance response, while *YUC2*/*5*/*8*/*9* genes were required for all shade avoidance responses (Fig 2; see Müller-Moulé et al., submitted, for a more detailed analysis). These differences could be explained by lower gene expression level of *TAA1* in leaves, in contrast of ubiquitous expression of *YUC2*/*5*/*8*/*9* (S5 Fig). Among shade-induced *AUX*/*IAA* family genes (see S2 Table), we tested the function of *IAA6*, and *IAA19*. The *iaa6*/*19* double showed a defect only in hypocotyl and leaf SAS, but not in acceleration of flowering time. Therefore *IAA6*/*IAA19* are required for response to shade in elongation. Similar to other auxin-related phenotypes, these shade-induced *AUX*/*IAA* genes likely function redundantly with other family members in the other two responses [83].

From transcriptome data it is not clear whether the auxin pathway is involved in acceleration of flowering time by shade, but phenotypic profiling indicates its involvement in flowering time control in response to shade. An auxin overproducing mutant (*atr4*) [84] and reduced auxin level mutant (*yuc2*/*5*/*8*/*9* and *sav3*/*taa1*) showed opposite flowering responses, supporting a role of auxin in this phenomenon (Fig 2).

How are light signaling and auxin pathways connected? All available data suggested that shade increases active auxin content by inducing auxin biosynthesis genes (*YUC*s) [80]. Among the *PIF*s, connection of *PIF4*/*5*/*7* and *SPT* to auxin has been reported [4,5,78,85,86,87]. At least some SAS traits are reduced in single or multiple *pif4*/*5*/*7* mutants (summarized in S3 Table). In our study, *yuc2*/*5*/*8*/*9* mutant is defective in all three shade-avoidance responses, which is consistent with upregulation of *YUC2*/*5*/*8*/*9* genes by *PIF7* [5] and *YUC5*/*8*/*9* by *PIF5* [4]. Interaction of *TERMINAL FLOWER 2* (*TFL2*) protein with *IAA6* and *IAA19* could be another pathway to link SAS and auxin [88], although we were not able to test *TFL2* in this study. Nevertheless, *tfl2* showed reduced shade avoidance (EODFR response) in hypocotyl [88].

#### JA pathway

Evidence showing a link between light and JA pathways has been accumulating for some time (reviewed in [89]). For example JA mediated defense responses are attenuated in shade [11]. This effect is mediated by COI1-JAZ10-dependent, salicylic acid-independent mechanisms [90]. Previous work also implicated JA signaling in *phyA* mediated responses to very low R:FR [48]. In tomato, stems of plants treated with shade for four days showed reduced JA levels [37]. Our transcriptome ORA with hormone responsive genes also shows shade-attenuation of the JA pathway (Table 3).

While it is known that shade attenuates JA responses, we used JA pathway mutants (Fig 1) to probe which JA components are required for SAS. Because *PHYA* is not important for the moderately low R/FR used in our study (see “light signaling pathway” section above) this enables us to determine whether JA is important for the typical *PHYB* mediated shade responses examined here. Our phenotypic profiling of these mutants showed that the JA pathway is involved in shade avoidance responses in leaf and flowering time, but we did not find significant effects on hypocotyl response (Fig 2, and S3 Table). This is consistent with GO analysis of transcriptomes of hypocotyl and juvenile plants (Table 1, 2, and 4) where JA GO categories were over-represented in the leaf/apical region but not in the hypocotyl data set. Reduced JA mediated plant immunity by shade is found in adult plants, but it is not known if this is true for hypocotyl. Comparison of shade-responsive genes between hypocotyl and leaf/apical region and comparison of phenotypic profiling predict that JA mediated plant immunity in hypocotyl is not affected by shade. In conclusion, we found that interaction of JA pathways and SAS network were not unidirectional but bidirectional.

Interestingly, partially different subsets of JA pathway genes are required for normal leaf and flowering shade response: *COI1* and *JASMONATE RESISTANT 1* (*JAR1*) for leaf response and *COI1* and *JAZ5* for flowering time response. What might drive these differences? *JAR1* encodes a protein that catalyzes conjugation of Ile to JA to produce active JA, which is required for JA mediated immunity [89]. Therefore one possibility is that JA-Ile is the active component for leaf response while other active JA-related compounds such as OPDA or cis-jasmone [89], could be the active components for flowering time. Another possibility is that *JAZ5* acts in a flowering time specific pathway.

It is interesting that JA biosynthesis mutants (*allene oxide synthase* (*aos*) and *12-oxophytodienoate-reductase 3* (*opr3*)) still retained weak shade-avoidance responses although a JA receptor mutant (*coi1-16*) had a defect of shade avoidance responses in petiole and flowering time (Fig 2). The fact that *opr3* mutants retaining JA biosynthesis in certain condition [91] may explain this. Alternatively, native JA related compounds released from neighbor plants in our experiments might partially rescue our JA biosynthesis mutants.

*MYC2* is a basic helix-loop-helix (bHLH) TF important for JA mediated immune responses that acts redundantly with its homologs, *MYC3* and *MYC4* [92]. Mutation in *MYC2* slightly reduced SAS in adult plants (Fig 2, and S3 Table), raising the possibility that the *MYC2*/*3*/*4* redundancy is also true for SAS. *PHYTOCHROME AND FLOWERING TIME 1* (*PFT1*) encodes the conserved MED25 subunit of the mediator complex, which is involved in JA-mediated immune system as well as light-signaling pathways [46,93,94,95]. Our hypothesis that *pft1* mutant impairs SAS was not confirmed because *pft1* did not show detectable differences compared with Col in any phenotypes we tested.

Our phenotypic profiling data showed less contribution of JA related-genes to shade-induced hypocotyl elongation than expected from previous data. For example, it was previously reported that exogenous MeJA application inhibited hypocotyl growth [96] and that this effect was influenced by *PHYB* [97]. Another examples is that *coi1* mutants showed an increases response to low R/FR [48]. The inconsistency with our results could be due to differences between exogenous JA application in the previous study versus the use of JA pathway mutants in our study, or due to differences in light conditions: previous studies used continuous monochromatic red light or extremely low R/FR (0.068), while our study used low R/FR (0.5). Therefore how light quality influences JA-dependent SAS is of future interest.

How JA pathways modulate petiole elongation is also unclear. Possible mechanism is promotion of PIF activities by binding of JAZ proteins to DELLA proteins that suppress PIF protein activities) [98]. Another link between JA and growth is that exogenous MeJA delays the start of endoreduplication cycle [99]. Interaction of these factors is of future interest.

#### Gibberellic acid pathways

The GA pathway is involved in shade-induced hypocotyl and petiole elongation as shown by GA biosynthesis inhibitor treatments and a GA deficient mutant (*ga requiring 1* (*ga1-3*)) [8]. The GA signaling *DELLA* gene family is reported to have a weak effect in hypocotyl SAS and none in petiole SAS [8]. We used another GA deficient mutant line (*ga20ox1*/*2*) [78,100] and a quadruple *DELLA* mutant line (*dellaQ*) [101] to test if GA is involved in other SAS traits (Fig 2). Similar to *ga1-3*, *ga20ox1*/*2* showed reduced response to shade in hypocotyl (Fig 2). In contrast to *ga1-3*, *ga20ox1*/*2* showed shade-induced petiole elongation even though both organs were about 60% of wild-type size (S3A and S3B Fig). In agreement of previous studies, *dellaQ* showed very weak influence on shade avoidance responses of hypocotyl and petioles in our condition (Fig 2, S3A, and S3B Fig). Both GA deficient and GA signaling mutants showed normal shade response for flowering time (Fig 2 and S3 Table), suggesting that GA pathway is not involved in flowering time acceleration by shade.

#### Circadian clock pathways

We found that over-expression of *RVE8*, a circadian clock component, caused moderately exaggerated shade responses in flowering time (Fig 2). This is consistent with the finding that natural variation of *ELF3* modulates flowering time response to shade [44]. Recent studies showed direct targets of *RVE8* TF [102]. Among them *PSEUDO RESPONSE REGULATOR*5 (*PRR5*), another circadian clock component, is of interest because *PRR5* is activated by RVE8 [102] and knocking out *PRR5* caused exaggerated shade avoidance response in petiole possibly through elevation of *PIF4* and *PIF5* expression levels [103]. Also it has been shown that circadian modulation of hypocotyl response to shade is mediated by circadian clock regulation of *PIL1* expression [30]. However, our shade avoidance assay for flowering time showed *pif4*/*5* and *pil1* had normal flowering time shade responses. It would be interesting to test if a *RVE8*—*PRR5* pathway contributes to shade response in flowering time.

#### Novel component

Our mutant analysis defined a new components of shade avoidance responses; a potassium channel gene (*POTASSIUM CHANNEL IN ARABIDOPSIS THALIANA 1*, *KAT1*). The *KAT1* gene encodes a potassium channel gene, which is locally expressed in guard cells [104,105]. *KAT1* potassium channel is inactivated upon ABA treatment and mediates stomata closure [106]. *KAT1* is thought to be important for vegetative growth [107], but its molecular mechanisms are unknown. Recently ABA was shown to be involved in suppression of branching by shade [36]. It is not clear if ABA pathways were involved in shade avoidance responses described in this paper, although ABA responsive genes were enriched in shade responsive genes (see above). Interestingly *KAT1* gene expression is induced by auxin [108,109], which may be related to its role in petiole SAS.

### Phenotypic-clustered SAS mutants may share common functions

In *C*. *elegans*, multiple phenotypic profiling showed that mutants with similar phenotypic profiles function in shared pathways [16]. In our phenotypic clusters (Fig 2), two flowering time mutant lines (*gi-2* and *co-9*) cluster together. These are known from prior studies to act in photoperiodic induction of flowering, and to have reduced shade response for flowering time but not petiole elongation [42] (cluster 7), showing proof of concept. In our data, clusters that consist of SAS mutants are of particular interest (clusters 9, 10, 11, and 12) because they could indicate shared membership in sub-networks of the shade avoidance pathway. Therefore, the following genes likely function in common sub-networks: *IAA6*, *IAA19*, *PIL1*, *LZF1*, *HY5*, and *SPT* in cluster 9 (hyp and pet), and *PAR1*, *KAT1*, *PIF4*, *PIF5*, *MIDA9*, *PIF3*, and *JAR1* in cluster 10 (pet only). Opposite effects of mutations were found in cluster 12; mutation caused reduced responses in hypocotyl and petiole, while its caused exaggerated responses in flowering time. Molecular networks within these clusters are of future interests.

### Mutants affecting of flowering time regulation under simulated sun

We observed many genotypes that showed altered flowering time in sun condition (S6 Fig). In this section, we will discuss about known components and then novel components in flowering time pathways.

#### Known components

As reported known knock out of master regulators of flowering time (*CO* and *GI*) showed late flowering in our long-day condition [110,111,112]. Light signaling has been known to modulate flowering time [113]. Phytochromes affect flowering time by post-transcriptional regulation and cryptochromes affect flowering time by both transcriptional and post-transcriptional regulation [62]. We observed that *phyB*, *phyA*/*B*, and *cry1*/*2* mutants showed flowering time phenotype as reported. Early flowering phenotype of *hy5 hy5 homolog* (*hyh*) is also reported [114]. *PIF4* has been known to induce a master regulator of flowering time, *FT* gene, upon raised temperature [115]. *pif4*/*5* mutant showed late flowering time, so that it is likely that PIF4 and/or PIF5 induce *FT* gene expression in our condition.

GA is essential for flowering time control. The GA biosynthesis double mutant (*ga20ox*1/*2*) showed delayed flowering in our experiment, consistent with earlier reports [100]. However, this mutant showed normal shade effects on flowering time (see above).

The circadian clock is also important for flowering time control. As reported, we found opposite phenotypes of *rve8* (early flowering) and *RVE8*-OX (late flowering) [116].

#### New components

It is surprising that auxin was involved in flowering time regulation, since it is not incorporated into current models of flowering time pathways (reviewed in [113]). However, exogenous auxin has been reported to delay flowering time, perhaps due to induced damage on plants [117]. This result is consistent with our auxin biosynthesis mutant flowering time data; overproduction delayed flowering time (*atr4*) while reduced production accelerated flowering time (*yuc2*/*5*/*8*/*9* and *taa1*). These results suggest that it is necessary to reinvestigate the involvement of auxin pathways in flowering time.

It has been shown that *coi1* mutants flowered early [48,98]. We observed that other JA mutants also show altered flowering time. Not only JA biosynthesis mutants (*aos* and *opr3*) and JA receptor mutant (*coi1-16*), but also three JA signaling component mutants (*JAZ1* RNAi, *jaz5-1*, *myc2*) showed early flowering phenotypes under sun condition. Early flowering of *myc2* is inconsistent with a previous report showing its late flowering phenotype [118] possibly due to different photoperiods. It will be interesting to investigate how JA pathways regulate flowering time. One possibility is that JA pathways interact with GA pathways in control of flowering, similar to their interaction in growth control [98].

Our data showed that two SPT knock out mutants (*spt-11* and *spt-12*), *PAR1* RNAi, *sth2*, and two KAT1 knock out mutants (*kat1-1* and *kat1-2*) showed early flowering phenotype, while *SCL13* anti-sense line 1 (*scl13* as1) and *hfr1* showed late flowering phenotype. Early flowering of *spt-11* has been reported [119] and that was confirmed by another allele (*spt-12*) in our study. Early flowering phenotype of *PAR1* RNAi, *sth2*, and *kat1-1* have not been previously reported and their connections to the flowering time pathway are unknown.

### Conclusion

Here we showed that RNA-seq followed by phenotypic profiling is a powerful approach for elucidating complex SAS pathways and discovery of new SAS components. A similar approach was successful in searching new components of de-etiolation of seedlings, a developmental stage with a simple architecture [24]. Our study expanded this approach to show the transcriptome-based discovery of new mutants are also effective for complex syndrome by multiple phonotypic profiling.

After our phenotypic profiling, an additional SAS mutant line has been reported which contained genes that were also in our shade-responsive genes. Specifically, we found that *BR ENHANCED EXPRESSION 3* (*BEE3*), a bHLH TF, was induced by shade (S2 Table). It was recently showed that the *bee1 bee2 bee3* triple mutant has altered hypocotyl SAS [120], perhaps due to the altered BR signaling in this triple [121]. This example is additional evidence that our strategy is effective to find novel SAS mutants. Further analysis is needed for elucidating interactions between these genes and/or pathways. Our approaches are straightforward and cost effective, so that these should be applicable to other cases in general.

We found that the effects of some mutations were context-dependent (only found for some organs or developmental stages) whereas others were ubiquitous. Those mutations that affect all organs points to shared mechanisms underlying the SAS in different organs. The mutations that have context-dependent effects could indicate unique genes functioning in the different organs or more quantitative differences in the relative importance of the components in different organs. Regardless the fact that we did find organ-specific effects suggests that we need to be cautious when generalizing conclusions from hypocotyl studies.

## Materials and Methods

### Light condition

For simulated sun condition, white light (cool-white fluorescent light) was supplemented with far-red light (provided by LEDs (Orbitec, inc) to obtain R/FR = 1.86. For simulated shade condition, white light was supplemented with far-red LEDS to obtain R/FR = 0.52. Both condition had 80–100 μE of Photosynthetically Active Radiation (PAR). Plants were grown under long day condition (16 hour light/8 hour dark) at constant temperature (22°C). For hypocotyl experiments, seedlings were grown under simulated sun (R/FR = 1.3) or simulated shade condition (R/FR = 0.5) with combination of LED lights (Quantum Devices Snap-Lite) [122]. Ambient light spectrum was measured by Black-Comet (StellarNet, Florida).

### Plant materials

*Arabidopsis* seed stocks used in this study are listed in Fig 1. To confirm genotypes of T-DNA insertion lines ordered from Arabidopsis Biological Resource Center (ABRC), genomic DNA was extracted (DNeasy Plant Mini kit, Qiagen) and subject to genomic PCR. cDNA was synthesized by direct mRNA extraction [123] and quantitative PCR (qPCR) was done with homemade SYBR green master mix with the iCycler Multicolor real-time PCR detection system (Bio-Rad). For *kat1* mutants, standard RT-PCR was done. Primers used for genomic PCR and (q)RT-PCR and their results were summarized in S4 Table. *Arabidopsis* seeds were imbibed with water on filter papers and stored them at 4°C for four days. Three days after stratification under sun condition, three germinated seeds were transferred to soil in a well of 5x10 well flat. Fourteen days after stratification, excess seedlings were removed to leave one well-grown plant per pot and the flats were transferred to either sun or shade condition. For hypocotyl growth measurements, seeds were grown on vertical square plates [124] with 1/2 MSMO, 5 mM 2-(N-morpholino)ethanesulfonic acid (MES, pH = 5.8, Sigma), and 0.8% agar (Sigma). Each plate was divided into three rows and two columns and in six spaces five or six seeds of six genotypes were sown. Genotype positions were randomized in repeated sets. 4593 seedling images were taken by a scanner and hypocotyl length was measured by ImageJ (http://rsb.info.nih.gov/ij/) [125].

### RNA-seq library preparation and sequencing

For RNA extraction, plants were treated with shade starting at ZT 4 or left in the sun. We prepared two replicates of each sample at 1 hour and 4 hours after sun and shade treatment and five plants were pooled for each replicate. Cotyledons, hypocotyls, and roots were removed from the samples, leaving leaves and apical tissue. Total RNA from the plants was extracted using RNeasy Plant Mini kit (Qiagen) with DNAse treatment (Qiagen). Five μg total RNA was used to construct mRNA library using mRNA-Seq-8 sample Prep kit (Illumina). The resulting cDNA libraries were sequenced by Illumina GAIIx with 40 bp single end mode. Basic statistics of mapping results are given in S1 Table.

### Differential expression analysis and over-representation analysis (ORA)

Reads after sorting according to barcodes were subjected to removal of adaptor contamination by custom Perl scripts. Reads were mapped by TopHat [126] to *Arabidopsis* reference genome using known annotation (TAIR10). Differentially expressed genes were extracted by edgeR package [127] in R statistical environment [128] (FDR <0.001). ORA was done by GOseq package [129] in R statistical environment. GO analysis was done by using GO category database package from Bioconductor (org.At.tair.db and ANNOTATE package). For ORA of hormone responsive genes custom categories were used as defined in Supplemental Table S9 in [130] and Supplemental S1 Table in [131]. GO analysis of shade-responsive genes in hypocotyl [7] was done using the GO Web site (http://amigo.geneontology.org/cgi-bin/amigo/term_enrichment; [132]).

### Phenotype measurement and analysis

For scoring leaf phenotypes, 26 day old plants were dissected and leaf images were recorded by a flatbed scanner (Epson, Perfection V700 PHOTO). Scanned images were measured using ImageJ [125] and the LeafJ plugin [25] to determine petiole length, leaf blade length, leaf blade width, and leaf blade area. Days to bolting was scored to measure flowering time. Leaf phenotypes (petiole length, leaf blade length, leaf blade width, leaf blade area) were measured from 10 sets of experiments with 1268 plants in total. For flowering time (days to bolting) measurement, 1950 plants were measured in total. Each phenotype was fitted by mixed effects model, i.e.

trait=plant+treatment+plant:treatment+(treatment|set)+ε

where plant is a mutant/overexpressor, treatment is sun or shade condition, plant:treatment is interaction of “plant” and “treatment”, (treatment|set) is the random effect associated with the treatment in set of experiments, and ε is the error. The model was applied to each trait to calculate coefficient (“sun” value). For leaf traits where we measured across multiple leaves (from leaf 3 to leaf 6) for a given trait we treated leaf as a random effect, using the following model

trait=plant+treatment+plant:treatment+(1|leaf)+(treatment|set)+ε

Mutants were considered to have a defect in SAS when the plant:treatment term was significant (P<0.05), indicating that the genotype of the plant (mutant versus wild-type) affected the response to shade.

For flowering time, days to bolting was log2 transformed. We found that acceleration of flowering time by shade treatment was strongly correlated with days to bolting in sun condition, i.e., late flowering mutants had more shade-accelerated flowering time than Col (S2G Fig). To address this issue we regressed flowering time shade response on average sun flowering time for each genotype and calculated the residuals from the regression [122]. These residuals represent the amount of flowering time shade response that was not predicted by the sun flowering time. The residuals for shade treated plants were then used in the mixed effects model (S3H Fig).

residuals=plant+(1|set)+ ε

The lme4 (R package version 1.0–6) [133] and lmerTest [134] packages in R was used for these analyses. All phenotyping data is summarized in S5 Table.

Heatmaps for phenotypic clustering were drawn after scaling each trait data and centered at Col.

All R scripts for this paper and raw data are available at https://bitbucket.org/knozue/sasphenotyping.

### Accession numbers

RNA-seq data in this study have been deposited in the NCBI SRA (Study ID PRJNA214254) and the NCBI GEO database (accession GSE66967). Mutants used in this study are listed in Fig 1.

## Supporting Information

S1 Fig Comparison of different platform and tissues with fold-changes of shade-responsive gene expression. Current RNA-seq data (juvenile plants under low R:FR labeled as “Nozue”) and microarray (leaf or petiole treated with EODFR [6] labeled as “Kozuka”), and hypocotyl treated with low R:FR (1 hour [7,26] (labeled as “Tao” and “Sessa.1h”, 4 days [26] labeled as “Sessa.4d”)).
(PDF)

S2 Fig Model checking for each trait. (A) hypocotyl, (B) petiole length, (C) leaf blade length, (D) leaf blade width, (E) leaf blade area, (F) petiole length/leaf blade length ratio, (G) flowering time, and (H) flowering time (log2 transformed).
(PDF)

S3 Fig Graphs of each trait. (A) hypocotyl, (B) petiole length, (C) leaf blade length, (D) leaf blade width, (E) leaf blade area, (F) petiole length/leaf blade length ratio, (G) flowering time (log2 transformed) and (H) flowering time (log2 transformed residuals). Error bars in (A) to (G) represent standard errors. Genotype names in (H) indicate lines whose flowering time shade response differs significantly from prediction by regression (p < 0.05).
(PDF)

S4 Fig Col and *pif3-3* petiole length. Plants were grown in simulated sun and shade using our standard conditions. Three independent experiments were performed and a total of 20 to 41 plants were examined per treatment/genotype combination. *pif3-3* has a significantly reduced response to shade (p<0.01 for genotype X treatment interaction in linear regression).
(PDF)

S5 Fig Expression pattern of *TAA1* and *YUC2*/*5*/*8*/*9*. Developmental expression pattern was obtained from eFP browser [136].
(TIF)

S6 Fig Heatmap with absolute values. Values were normalized and centered on Col (i.e., Col value = 0) and visualized with color coding (magenta indicates larger value than Col while green indicates smaller value relative to Col). Colors of asterisks indicate genetic background of each mutant, i.e., Col (white), Ws (yellow), and L*er* (light blue).
(TIF)

S1 Table Statistics of RNA-seq data. In each condition two biological replicates are shown in a and b.
(XLSX)

S2 Table A complete list of shade-responsive genes in leaf/apical region of plants. Blue letters; shade-induced genes, pink letters; shade-repressed genes, yellow box; no probes on ATH1 microarray. Bold text; known shade-responsive genes. Comparison with transcriptome data from EODFR treated leaf blade and petiole [5] are also shown.
(XLSX)

S3 Table Summary of SAS phenotypes with known SAS mutants and mutants used in this study. (XLSX)

S4 Table List of primers used in genotyping and (q)RT-PCR and their results. (Nearly) homozygous lines are shown in light blue.
(XLSX)

S5 Table Complete phenotype data after applying mixed effects models. (CSV)

## References

[1] Casal JJ (2013) Photoreceptor signaling networks in plant responses to shade. Annu Rev Plant Biol 64: 403–427. 10.1146/annurev-arplant-050312-120221 23373700

[2] Ballaré CL, Sánchez RA, Scopel AL, Casal JJ, Ghersa CM (1987) Early detection of neighbour plants by phytochrome perception of spectral changes in reflected sunlight. Plant Cell Environ 10: 551–557.

[3] Lorrain S, Allen T, Duek PD, Whitelam GC, Fankhauser C (2008) Phytochrome-mediated inhibition of shade avoidance involves degradation of growth-promoting bHLH transcription factors. Plant J 53: 312–323. 18047474 10.1111/j.1365-313X.2007.03341.x

[4] Hornitschek P, Kohnen MV, Lorrain S, Rougemont J, Ljung K, et al (2012) Phytochrome interacting factors 4 and 5 control seedling growth in changing light conditions by directly controlling auxin signaling. Plant J 71: 699–711. 10.1111/j.1365-313X.2012.05033.x 22536829

[5] Li L, Ljung K, Breton G, Schmitz RJ, Pruneda-Paz J, et al (2012) Linking photoreceptor excitation to changes in plant architecture. Genes Dev 26: 785–790. 10.1101/gad.187849.112 22508725 PMC3337452

[6] Kozuka T, Kobayashi J, Horiguchi G, Demura T, Sakakibara H, et al (2010) Involvement of auxin and brassinosteroid in the regulation of petiole elongation under the shade. Plant Physiol 153: 1608–1618. 10.1104/pp.110.156802 20538889 PMC2923899

[7] Tao Y, Ferrer JL, Ljung K, Pojer F, Hong F, et al (2008) Rapid synthesis of auxin via a new tryptophan-dependent pathway is required for shade avoidance in plants. Cell 133: 164–176. 10.1016/j.cell.2008.01.049 18394996 PMC2442466

[8] Djakovic-Petrovic T, Wit Md, Voesenek LACJ, Pierik R (2007) DELLA protein function in growth responses to canopy signals. Plant J 51: 117–126. 17488236 10.1111/j.1365-313X.2007.03122.x

[9] Kurepin LV, Emery RJN, Pharis RP, Reid DM (2007) The interaction of light quality and irradiance with gibberellins, cytokinins and auxin in regulating growth of *Helianthus annuus* hypocotyls. Plant Cell Environ 30: 147–155. 17238906 10.1111/j.1365-3040.2006.01612.x

[10] Carabelli M, Possenti M, Sessa G, Ciolfi A, Sassi M, et al (2007) Canopy shade causes a rapid and transient arrest in leaf development through auxin-induced cytokinin oxidase activity. Genes Dev 21: 1863–1868. 17671088 10.1101/gad.432607 PMC1935025

[11] Moreno JE, Tao Y, Chory J, Ballare CL (2009) Ecological modulation of plant defense via phytochrome control of jasmonate sensitivity. Proc Natl Acad Sci U S A 106: 4935–4940. 10.1073/pnas.0900701106 19251652 PMC2660767

[12] Kegge W, Weldegergis BT, Soler R, Vergeer-Van Eijk M, Dicke M, et al (2013) Canopy light cues affect emission of constitutive and methyl jasmonate-induced volatile organic compounds in *Arabidopsis thaliana* . New Phytol 200: 861–874. 10.1111/nph.12407 23845065 PMC4283982

[13] Sozzani R, Benfey P (2011) High-throughput phenotyping of multicellular organisms: finding the link between genotype and phenotype. Genome Biol 12: 219 10.1186/gb-2011-12-3-219 21457493 PMC3129668

[14] Winzeler EA, Shoemaker DD, Astromoff A, Liang H, Anderson K, et al (1999) Functional characterization of the *S*. *cerevisiae* genome by gene deletion and parallel analysis. Science 285: 901–906. 10436161 10.1126/science.285.5429.901

[15] Neumann B, Walter T, Heriche JK, Bulkescher J, Erfle H, et al (2010) Phenotypic profiling of the human genome by time-lapse microscopy reveals cell division genes. Nature 464: 721–727. 10.1038/nature08869 20360735 PMC3108885

[16] Green RA, Kao H-L, Audhya A, Arur S, Mayers JR, et al (2011) A high-resolution *C*.*elegans* essential gene network based on phenotypic profiling of a complex tissue. Cell 145: 470–482. 10.1016/j.cell.2011.03.037 21529718 PMC3086541

[17] Yanik MF, Rohde CB, Pardo-Martin C (2011) Technologies for micromanipulating, imaging, and phenotyping small invertebrates and vertebrates. Annu Rev Biomed Eng 13: 185–217. 10.1146/annurev-bioeng-071910-124703 21756142

[18] Atwell S, Huang YS, Vilhjalmsson BJ, Willems G, Horton M, et al (2010) Genome-wide association study of 107 phenotypes in *Arabidopsis thaliana* inbred lines. Nature 465: 627–631. 10.1038/nature08800 20336072 PMC3023908

[19] Berger B, Regt B, Tester M (2012) High-throughput phenotyping of plant shoots In: Normanly J, editor. High-throughput phenotyping in plants: Humana Press pp. 9–20. 10.1007/978-1-61779-995-2_2 22893282

[20] Spalding EP, Miller ND (2013) Image analysis is driving a renaissance in growth measurement. Curr Opin Plant Biol 16: 100–104. 10.1016/j.pbi.2013.01.001 23352714

[21] Dornbusch T, Lorrain S, Kuznetsov D, Fortier A, Liechti R, et al (2012) Measuring the diurnal pattern of leaf hyponasty and growth in *Arabidopsis*—a novel phenotyping approach using laser scanning. Funct Plant Biol 39: 860–869. 10.1071/FP12018 32480836

[22] Bruex A, Kainkaryam RM, Wieckowski Y, Kang YH, Bernhardt C, et al (2012) A gene regulatory network for root epidermis cell differentiation in *Arabidopsis* . PLoS Genet 8: e1002446 10.1371/journal.pgen.1002446 22253603 PMC3257299

[23] Sentandreu M, Martin G, Gonzalez-Schain N, Leivar P, Soy J, et al (2011) Functional profiling identifies genes involved in organ-specific branches of the PIF3 regulatory network in *Arabidopsis* . Plant Cell 23: 3974–3991. 10.1105/tpc.111.088161 22108407 PMC3246323

[24] Khanna R, Shen Y, Toledo-Ortiz G, Kikis EA, Johannesson H, et al (2006) Functional profiling reveals that only a small number of phytochrome-regulated early-response genes in *Arabidopsis* are necessary for optimal deetiolation. Plant Cell 18: 2157–2171. 16891401

[25] Maloof JN, Nozue K, Mumbach MR, Palmer CM (2013) LeafJ: An ImageJ plugin for semi-automated leaf shape measurement. J Vis Exp 71: e50028. 10.3791/50028 PMC3582691 23380664

[26] Sessa G, Carabelli M, Sassi M, Ciolfi A, Possenti M, et al (2005) A dynamic balance between gene activation and repression regulates the shade avoidance response in *Arabidopsis* . Genes Dev 19: 2811–2815. 16322556 10.1101/gad.364005 PMC1315388

[27] Carabelli M, Morelli G, Whitelam G, Ruberti I (1996) Twilight-zone and canopy shade induction of the Athb-2 homeobox gene in green plants. Proc Natl Acad Sci U S A 93: 3530–3535. 11607652 10.1073/pnas.93.8.3530 PMC39644

[28] Roig-Villanova I, Bou-Torrent J, Galstyan A, Carretero-Paulet L, Portoles S, et al (2007) Interaction of shade avoidance and auxin responses: a role for two novel atypical bHLH proteins. EMBO J 26: 4756–4767. 17948056 10.1038/sj.emboj.7601890 PMC2080812

[29] Sorin C, Salla-Martret M, Bou-Torrent J, Irma Roig-Villanova I, MartÌnez-GarcÌa J (2009) ATHB4, a regulator of shade avoidance, modulates hormone response in *Arabidopsis* seedlings. Plant J 59: 266–277. 10.1111/j.1365-313X.2009.03866.x 19392702

[30] Salter MG, Franklin KA, Whitelam GC (2003) Gating of the rapid shade-avoidance response by the circadian clock in plants. Nature 426: 680–683. 14668869 10.1038/nature02174

[31] Finlayson SA, Lee IJ, Mullet JE, Morgan PW (1999) The mechanism of rhythmic ethylene production in sorghum. The role of phytochrome B and simulated shading. Plant Physiol 119: 1083–1089. 10.1104/pp.119.3.1083 PMC32090 10069847

[32] Pierik R, Cuppens MLC, Voesenek LACJ, Visser EJW (2004) Interactions between ethylene and gibberellins in phytochrome-mediated shade avoidance responses in tobacco. Plant Physiol 136: 2928–2936. 15448197 10.1104/pp.104.045120 PMC523355

[33] Millenaar FF, Cox MC, van Berkel YE, Welschen RA, Pierik R, et al (2005) Ethylene-induced differential growth of petioles in Arabidopsis. Analyzing natural variation, response kinetics, and regulation. Plant Physiol 137: 998–1008. 15728343 10.1104/pp.104.053967 PMC1065400

[34] Smalle J, Haegman M, Kurepa J, Van Montagu M, Straeten DVD (1997) Ethylene can stimulate *Arabidopsis* hypocotyl elongation in the light. Proc Natl Acad Sci U S A 94: 2756–2761. 11038610 10.1073/pnas.94.6.2756 PMC20163

[35] Pierik R, Whitelam GC, Voesenek LACJ, de Kroon H, Visser EJW (2004) Canopy studies on ethylene-insensitive tobacco identify ethylene as a novel element in blue light and plant-plant signalling. Plant J 38: 310–319. 15078333 10.1111/j.1365-313X.2004.02044.x

[36] Reddy SK, Holalu SV, Casal JJ, Finlayson SA (2013) Abscisic acid regulates axillary bud outgrowth responses to the ratio of red to far-red light. Plant Physiol 163: 1047–1058. 10.1104/pp.113.221895 23929720 PMC3793024

[37] Cagnola J, Ploschuk E, Benech-Arnold T, Finlayson S, Casal JJ (2012) Stem transcriptome reveals mechanisms to reduce the energetic cost of shade-avoidance responses in tomato. Plant Physiol 160: 1110–1119. 10.1104/pp.112.201921 22872775 PMC3461533

[38] Nagatani A, Chory J, Furuya M (1991) Phytochrome B is not detectable in the *hy3* mutant of *Arabidopsis*, which Is deficient in responding to end-of-day far-red light treatments. Plant Cell Physiol 32: 1119–1122.

[39] Reed JW, Nagpal P, Poole DS, Furuya M, Chory J (1993) Mutations in the gene for the red/far-red light receptor phytochrome B alter cell elongation and physiological responses throughout *Arabidopsis* development. Plant Cell 5: 147–157. 8453299 10.1105/tpc.5.2.147 PMC160258

[40] Robson P, Whitelam GC, Smith H (1993) Selected components of the shade-avoidance syndrome are displayed in a normal manner in mutants of *Arabidopsis thaliana* and *Brassica rapa* deficient in phytochrome B. Plant Physiol 102: 1179–1184. 12231894 10.1104/pp.102.4.1179 PMC158903

[41] Devlin PF, Robson PR, Patel SR, Goosey L, Sharrock RA, et al (1999) Phytochrome D acts in the shade-avoidance syndrome in *Arabidopsis* by controlling elongation growth and flowering time. Plant Physiol 119: 909–915. 10069829 10.1104/pp.119.3.909 PMC32105

[42] Wollenberg AC, Strasser B, Cerdan PD, Amasino RM (2008) Acceleration of flowering during shade avoidance in *Arabidopsis* alters the balance between FLOWERING LOCUS C-mediated repression and photoperiodic induction of flowering. Plant Physiol 148: 1681–1694. 10.1104/pp.108.125468 18790998 PMC2577263

[43] Kim SY, Yu X, Michaels SD (2008) Regulation of CONSTANS and FLOWERING LOCUS T expression in response to changing light quality. Plant Physiol 148: 269–279. 10.1104/pp.108.122606 18667727 PMC2528114

[44] Jimenez-Gomez JM, Wallace AD, Maloof JN (2010) Network analysis identifies ELF3 as a QTL for the shade avoidance response in Arabidopsis. PLoS Genet 6: e1001100 10.1371/journal.pgen.1001100 20838594 PMC2936530

[45] Kanyuka K, Praekelt U, Franklin KA, Billingham OE, Hooley R, et al (2003) Mutations in the huge *Arabidopsis* gene *BIG* affect a range of hormone and light responses. Plant J 35: 57–70. 12834402 10.1046/j.1365-313x.2003.01779.x

[46] Cerdan PD, Chory J (2003) Regulation of flowering time by light quality. Nature 423: 881–885. 12815435 10.1038/nature01636

[47] Franklin KA, Praekelt U, Stoddart WM, Billingham OE, Halliday KJ, et al (2003) Phytochromes B, D, and E act redundantly to control multiple physiological responses in *Arabidopsis* . Plant Physiol 131: 1340–1346. 12644683 10.1104/pp.102.015487 PMC166893

[48] Robson F, Okamoto H, Patrick E, Harris SR, Wasternack C, et al (2010) Jasmonate and phytochrome A signaling in *Arabidopsis* wound and shade responses are integrated through JAZ1 stability. Plant Cell 22: 1143–1160. 10.1105/tpc.109.067728 20435902 PMC2879735

[49] Steindler C, Matteucci A, Sessa G, Weimar T, Ohgishi M, et al (1999) Shade avoidance responses are mediated by the ATHB-2 HD-zip protein, a negative regulator of gene expression. Development 126: 4235–4245. 10477292 10.1242/dev.126.19.4235

[50] Rolauffs S, Fackendahl P, Sahm J, Fiene G, Hoecker U (2012) *Arabidopsis* COP1 and SPA genes are essential for plant elongation but not for acceleration of flowering time in response to a low red light to far-red light ratio. Plant Physiol 160: 2015–2027. 10.1104/pp.112.207233 23093358 PMC3510128

[51] Franklin KA, Quail PH (2010) Phytochrome functions in *Arabidopsis* development. J Exp Bot 61: 11–24. 10.1093/jxb/erp304 19815685 PMC2800801

[52] Neff MM, Chory J (1998) Genetic interactions between phytochrome A, phytochrome B, and cryptochrome 1 during *Arabidopsis* development. Plant Physiol 118: 27–35. 9733523 10.1104/pp.118.1.27 PMC34865

[53] Casal JJ, Mazzella MA (1998) Conditional synergism between cryptochrome 1 and phytochrome B is shown by the analysis of *phyA*, *phyB*, and *hy4* simple, double, and triple mutants in *Arabidopsis* . Plant Physiol 118: 19–25. 9733522 10.1104/pp.118.1.19 PMC34855

[54] Casal JJ, Boccalandro H (1995) Co-action between phytochrome B and HY4 in *Arabidopsis thaliana* . Planta 197: 213–218. 8547813 10.1007/BF00202639

[55] Ahmad M, Cashmore AR (1997) The blue-light receptor cryptochrome 1 shows functional dependence on phytochrome A or phytochrome B in *Arabidopsis thaliana* . Plant J 11: 421–427. 9107032 10.1046/j.1365-313x.1997.11030421.x

[56] Hennig L, Funk M, Whitelam GC, Schafer E (1999) Functional interaction of cryptochrome 1 and phytochrome D. Plant J 20: 289–294. 10571889 10.1046/j.1365-313x.1999.t01-1-00599.x

[57] Castillon A, Shen H, Huq E (2009) Blue light induces degradation of the negative regulator phytochrome interacting factor 1 to promote photomorphogenic development of *Arabidopsis* seedlings. Genetics 182: 161–171. 10.1534/genetics.108.099887 19255368 PMC2674814

[58] Usami T, Matsushita T, Oka Y, Mochizuki N, Nagatani A (2007) Roles for the N- and C-terminal domains of phytochrome B in interactions between phytochrome B and cryptochrome signaling cascades. Plant Cell Physiol 48: 424–433. 17251203 10.1093/pcp/pcm012

[59] Usami T, Mochizuki N, Kondo M, Nishimura M, Nagatani A (2004) Cryptochromes and phytochromes synergistically regulate *Arabidopsis* root greening under blue light. Plant Cell Physiol 45: 1798–1808. 15653798 10.1093/pcp/pch205

[60] Meijer G, Engelsma G (1965) The synergistic influence of a pre-irradiation on the photoinhibitton of gherkin seedlings. Photochem Photobiol 4: 251–258.

[61] Hughes RM, Vrana JD, Song J, Tucker CL (2012) Light-dependent, dark-promoted interaction between *Arabidopsis* cryptochrome 1 and phytochrome B proteins. J Biol Chem 287: 22165–22172. 10.1074/jbc.M112.360545 22577138 PMC3381176

[62] Liu H, Liu B, Zhao C, Pepper M, Lin C (2011) The action mechanisms of plant cryptochromes. Trends Plant Sci 16: 684–691. 10.1016/j.tplants.2011.09.002 21983106 PMC3277817

[63] Crocco CD, Holm M, Yanovsky MJ, Botto JF (2010) AtBBX21 and COP1 genetically interact in the regulation of shade avoidance. Plant J 64: 551–562. 10.1111/j.1365-313X.2010.04360.x 21070414

[64] Pacín M, Legris M, Casal JJ (2013) COP1 re-accumulates in the nucleus under shade. Plant J 75: 631–641. 10.1111/tpj.12226 23647163

[65] Guo H, Yang H, Mockler TC, Lin C (1998) Regulation of flowering time by *Arabidopsis* photoreceptors. Science 279: 1360–1363. 9478898 10.1126/science.279.5355.1360

[66] Zuo Z, Liu H, Liu B, Liu X, Lin C (2011) Blue light-dependent interaction of CRY2 with SPA1 regulates COP1 activity and floral initiation in Arabidopsis. Curr Biol 21: 841–847. 10.1016/j.cub.2011.03.048 21514160 PMC3150455

[67] Liu Y, Li X, Li K, Liu H, Lin C (2013) Multiple bHLH proteins form heterodimers to mediate CRY2-dependent regulation of flowering-time in *Arabidopsis* . PLoS Genet 9: e1003861 10.1371/journal.pgen.1003861 24130508 PMC3794922

[68] Keller MM, Jaillais Y, Pedmale UV, Moreno JE, Chory J, et al (2011) Cryptochrome 1 and phytochrome B control shade-avoidance responses in *Arabidopsis* via partially independent hormonal cascades. Plant J 67: 195–207. 10.1111/j.1365-313X.2011.04598.x 21457375 PMC3135679

[69] Keuskamp DH, Sasidharan R, Vos I, Peeters AJ, Voesenek LA, et al (2011) Blue-light-mediated shade avoidance requires combined auxin and brassinosteroid action in *Arabidopsis* seedlings. Plant J 67: 208–217. 10.1111/j.1365-313X.2011.04597.x 21457374

[70] Ichihashi Y, Horiguchi G, Gleissberg S, Tsukaya H (2010) The bHLH transcription factor SPATULA controls final leaf size in *Arabidopsis thaliana* . Plant Cell Physiol 51: 252–261. 10.1093/pcp/pcp184 20040585

[71] Reymond MC, Brunoud G, Chauvet A, Martinez-Garcia JF, Martin-Magniette ML, et al (2012) A light-regulated genetic module was recruited to carpel development in *Arabidopsis* following a structural change to SPATULA. Plant Cell 24: 2812–2825. 10.1105/tpc.112.097915 22851763 PMC3426116

[72] Leivar P, Tepperman JM, Cohn MM, Monte E, Al-Sady B, et al (2012) Dynamic antagonism between phytochromes and PIF family basic helix-loop-helix factors induces selective reciprocal responses to light and shade in a rapidly responsive transcriptional network in *Arabidopsis* . Plant Cell 24: 1398–1419. 10.1105/tpc.112.095711 22517317 PMC3398554

[73] Soy J, Leivar P, Monte E (2014) PIF1 promotes phytochrome-regulated growth under photoperiodic conditions in *Arabidopsis* together with PIF3, PIF4, and PIF5. J Exp Bot 65: 2925–2936. 10.1093/jxb/ert465 24420574 PMC4056538

[74] Huq E, Al-Sady B, Hudson M, Kim C, Apel K, et al (2004) Phytochrome-interacting factor 1 is a critical bHLH regulator of chlorophyll biosynthesis. Science 305: 1937–1941. 15448264 10.1126/science.1099728

[75] Leivar P, Monte E (2014) PIFs: Systems Integrators in Plant Development. The Plant Cell Online 26: 56–78. 10.1105/tpc.113.120857 24481072 PMC3963594

[76] Monte E, Tepperman JM, Al-Sady B, Kaczorowski KA, Alonso JM, et al (2004) The phytochrome-interacting transcription factor, PIF3, acts early, selectively, and positively in light-induced chloroplast development. Proc Natl Acad Sci U S A 101: 16091–16098. 15505214 10.1073/pnas.0407107101 PMC528976

[77] Zhang Y, Mayba O, Pfeiffer A, Shi H, Tepperman JM, et al (2013) A quartet of PIF bHLH factors provides a transcriptionally centered signaling hub that regulates seedling morphogenesis through differential expression-patterning of shared target genes in Arabidopsis. PLoS Genet 9: e1003244 10.1371/journal.pgen.1003244 23382695 PMC3561105

[78] Nozue K, Harmer SL, Maloof JN (2011) Genomic analysis of circadian clock-, light-, and growth-correlated genes reveals PHYTOCHROME-INTERACTING FACTOR5 as a modulator of auxin signaling in *Arabidopsis* . Plant Physiol 156: 357–372. 10.1104/pp.111.172684 21430186 PMC3091056

[79] Spartz AK, Ren H, Park MY, Grandt KN, Lee SH, et al (2014) SAUR inhibition of PP2C-D phosphatases activates plasma membrane H+-ATPases to promote cell expansion in *Arabidopsis* . Plant Cell 26: 2129–2142. 24858935 10.1105/tpc.114.126037 PMC4079373

[80] de Wit M, Lorrain S, Fankhauser C (2014) Auxin-mediated plant architectural changes in response to shade and high temperature. Physiol Plant 151: 13–24. 10.1111/ppl.12099 24011166

[81] Mashiguchi K, Tanaka K, Sakai T, Sugawara S, Kawaide H, et al (2011) The main auxin biosynthesis pathway in *Arabidopsis* . Proc Natl Acad Sci U S A 108: 18512–18517. 10.1073/pnas.1108434108 22025724 PMC3215075

[82] Won C, Shen X, Mashiguchi K, Zheng Z, Dai X, et al (2011) Conversion of tryptophan to indole-3-acetic acid by *TRYPTOPHAN AMINOTRANSFERASES OF ARABIDOPSIS* and *YUCCA*s in *Arabidopsis* . Proc Natl Acad Sci U S A 108: 18518–18523. 10.1073/pnas.1108436108 22025721 PMC3215067

[83] Overvoorde PJ, Okushima Y, Alonso JM, Chan A, Chang C, et al (2005) Functional genomic analysis of the *AUXIN/INDOLE-3-ACETIC ACID* gene family members in *Arabidopsis thaliana* . Plant Cell 17: 3282–3300. 16284307 10.1105/tpc.105.036723 PMC1315369

[84] Delarue M, Prinsen E, Va H, Onckelen, Caboche M, et al (1998) *Sur2* mutations of *Arabidopsis thaliana* define a new locus involved in the control of auxin homeostasis. Plant J 14: 603–611. 9675903 10.1046/j.1365-313x.1998.00163.x

[85] Franklin KA, Lee SH, Patel D, Kumar SV, Spartz AK, et al (2011) Phytochrome-interacting factor 4 (PIF4) regulates auxin biosynthesis at high temperature. Proc Natl Acad Sci U S A 108: 20231–20235. 10.1073/pnas.1110682108 22123947 PMC3250122

[86] Nemhauser JL, Feldman LJ, Zambryski PC (2000) Auxin and ETTIN in *Arabidopsis* gynoecium morphogenesis. Development 127: 3877–3888. 10952886 10.1242/dev.127.18.3877

[87] Foreman J, White J, Graham I, Halliday K, Josse EM (2011) Shedding light on flower development: phytochrome B regulates gynoecium formation in association with the transcription factor *SPATULA* . Plant Signal Behav 6: 471–476. 21364315 10.4161/psb.6.4.14496 PMC3142372

[88] Valdes AE, Rizzardi K, Johannesson H, Para A, Sundas-Larsson A, et al (2011) *Arabidopsis thaliana TERMINAL FLOWER2* is involved in light-controlled signalling during seedling photomorphogenesis. Plant Cell Environ 35: 1013–1025. 10.1111/j.1365-3040.2011.02468.x 22145973

[89] Wasternack C, Hause B (2013) Jasmonates: biosynthesis, perception, signal transduction and action in plant stress response, growth and development. An update to the 2007 review in Annals of Botany. Ann Bot 111: 1021–1058. 10.1093/aob/mct067 23558912 PMC3662512

[90] Cerrudo I, Keller MM, Cargnel MD, Demkura PV, de Wit M, et al (2012) Low red/far-red ratios reduce *Arabidopsis* resistance to *Botrytis cinerea* and jasmonate responses via a COI1-JAZ10-dependent, salicylic acid-independent mechanism. Plant Physiol 158: 2042–2052. 10.1104/pp.112.193359 22371506 PMC3320205

[91] Chehab EW, Kim S, Savchenko T, Kliebenstein D, Dehesh K, et al (2011) Intronic T-DNA insertion renders *Arabidopsis opr3* a conditional jasmonic acid-producing mutant. Plant Physiol 156: 770–778. 10.1104/pp.111.174169 21487047 PMC3177274

[92] Fernandez-Calvo P, Chini A, Fernandez-Barbero G, Chico JM, Gimenez-Ibanez S, et al (2011) The *Arabidopsis* bHLH transcription factors MYC3 and MYC4 are targets of JAZ repressors and act additively with MYC2 in the activation of jasmonate responses. Plant Cell 23: 701–715. 10.1105/tpc.110.080788 21335373 PMC3077776

[93] Cevik V, Kidd BN, Zhang P, Hill C, Kiddle S, et al (2012) MEDIATOR25 acts as an integrative hub for the regulation of jasmonate-responsive gene expression in *Arabidopsis* . Plant Physiol 160: 541–555. 10.1104/pp.112.202697 22822211 PMC3440227

[94] Chen R, Jiang H, Li L, Zhai Q, Qi L, et al (2012) The *Arabidopsis* mediator subunit MED25 differentially regulates jasmonate and abscisic acid signaling through interacting with the MYC2 and ABI5 transcription factors. Plant Cell 24: 2898–2916. 10.1105/tpc.112.098277 22822206 PMC3426122

[95] Klose C, Buche C, Fernandez AP, Schafer E, Zwick E, et al (2012) The mediator complex subunit PFT1 interferes with COP1 and HY5 in the regulation of *Arabidopsis* light signaling. Plant Physiol 160: 289–307. 10.1104/pp.112.197319 22760208 PMC3440207

[96] Hou X, Lee LY, Xia K, Yan Y, Yu H (2010) DELLAs modulate jasmonate signaling via competitive binding to JAZs. Dev Cell 19: 884–894. 10.1016/j.devcel.2010.10.024 21145503

[97] Chen J, Sonobe K, Ogawa N, Masuda S, Nagatani A, et al (2013) Inhibition of arabidopsis hypocotyl elongation by jasmonates is enhanced under red light in phytochrome B dependent manner. J Plant Res 126: 161–168. 10.1007/s10265-012-0509-3 22825635 PMC3530149

[98] Yang D-L, Yao J, Mei C-S, Tong X-H, Zeng L-J, et al (2012) Plant hormone jasmonate prioritizes defense over growth by interfering with gibberellin signaling cascad. Proc Natl Acad Sci U S A 109: E1192–E1200. 10.1073/pnas.1201616109 22529386 PMC3358897

[99] Noir S, Bomer M, Takahashi N, Ishida T, Tsui TL, et al (2013) Jasmonate controls leaf growth by repressing cell proliferation and the onset of endoreduplication while maintaining a potential stand-by mode. Plant Physiol 161: 1930–1951. 10.1104/pp.113.214908 23439917 PMC3613466

[100] Rieu I, Ruiz-Rivero O, Fernandez-Garcia N, Griffiths J, Powers SJ, et al (2008) The gibberellin biosynthetic genes *AtGA20ox1* and *AtGA20ox2* act, partially redundantly, to promote growth and development throughout the *Arabidopsis* life cycle. Plant J 53: 488–504. 18069939 10.1111/j.1365-313X.2007.03356.x

[101] Achard P, Cheng H, De Grauwe L, Decat J, Schoutteten H, et al (2006) Integration of plant responses to environmentally activated phytohormonal signals. Science 311: 91–94. 16400150 10.1126/science.1118642

[102] Hsu PY, Devisetty UK, Harmer SL (2013) Accurate timekeeping is controlled by a cycling activator in *Arabidopsis* . eLife 2: e00473 10.7554/eLife.00473 23638299 PMC3639509

[103] Takase M, Mizoguchi T, Kozuka T, Tsukaya H (2013) The unique function of the Arabidopsis circadian clock gene PRR5 in the regulation of shade avoidance response. Plant Signal Behav 8: e23534 10.4161/psb.23534 23333981 PMC7030191

[104] Schachtman D, Schroeder J, Lucas W, Anderson J, Gaber R (1992) Expression of an inward-rectifying potassium channel by the *Arabidopsis* KAT1 cDNA. Science 258: 1654–1658. 8966547 10.1126/science.8966547

[105] Nakamura RL, McKendree WL Jr., Hirsch RE, Sedbrook JC, Gaber RF, et al (1995) Expression of an *Arabidopsis* potassium channel gene in guard cells. Plant Physiol 109: 371–374. 7480337 10.1104/pp.109.2.371 PMC157599

[106] Sutter J-U, Sieben C, Hartel A, Eisenach C, Thiel G, et al (2007) Abscisic acid triggers the endocytosis of the *Arabidopsis* KAT1 K+ channel and its recycling to the plasma membrane. Current Biol 17: 1396–1402. 17683934 10.1016/j.cub.2007.07.020

[107] Eisenach C, Chen Z-H, Grefen C, Blatt MR (2011) The trafficking protein SYP121 of *Arabidopsis* connects programmed stomatal closure and K+ channel activity with vegetative growth. Plant J 69: 241–251. 10.1111/j.1365-313X.2011.04786.x 21914010

[108] Abel S, Nguyen MD, Theologis A (1995) The PS-IAA4/5-like family of early auxin-inducible mRNAs in *Arabidopsis thaliana* . J Mol Biol 251: 533–549. 7658471 10.1006/jmbi.1995.0454

[109] Philippar K, Ivashikina N, Ache P, Christian M, Luthen H, et al (2004) Auxin activates *KAT1* and *KAT2*, two K+-channel genes expressed in seedlings of *Arabidopsis thaliana* . Plant J 37: 815–827. 14996216 10.1111/j.1365-313x.2003.02006.x

[110] Putterill J, Robson F, Lee K, Simon R, Coupland G (1995) The CONSTANS gene of *Arabidopsis* promotes flowering and encodes a protein showing similarities to zinc finger transcription factors. Cell 80: 847–857. 7697715 10.1016/0092-8674(95)90288-0

[111] Park DH, Somers DE, Kim YS, Choy YH, Lim HK, et al (1999) Control of circadian rhythms and photoperiodic flowering by the *Arabidopsis GIGANTEA* gene. Science 285: 1579–1582. 10477524 10.1126/science.285.5433.1579

[112] Fowler S, Lee K, Onouchi H, Samach A, Richardson K, et al (1999) *GIGANTEA*: a circadian clock-controlled gene that regulates photoperiodic flowering in *Arabidopsis* and encodes a protein with several possible membrane-spanning domains. EMBO J 18: 4679–4688. 10469647 10.1093/emboj/18.17.4679 PMC1171541

[113] Song YH, Ito S, Imaizumi T (2013) Flowering time regulation: photoperiod- and temperature-sensing in leaves. Trends Plant Sci 18: 575–583. 10.1016/j.tplants.2013.05.003 23790253 PMC3796012

[114] Holm M, Ma LG, Qu LJ, Deng XW (2002) Two interacting bZIP proteins are direct targets of COP1-mediated control of light-dependent gene expression in *Arabidopsis* . Genes Dev 16: 1247–1259. 12023303 10.1101/gad.969702 PMC186273

[115] Kumar SV, Lucyshyn D, Jaeger KE, Alos E, Alvey E, et al (2012) Transcription factor PIF4 controls the thermosensory activation of flowering. Nature 484: 242–245. 10.1038/nature10928 22437497 PMC4972390

[116] Rawat R, Takahashi N, Hsu PY, Jones MA, Schwartz J, et al (2011) REVEILLE8 and PSEUDO-REPONSE REGULATOR5 form a negative feedback loop within the *Arabidopsis* circadian clock. PLoS Genet 7: e1001350 10.1371/journal.pgen.1001350 21483796 PMC3069099

[117] Leopold AC (1958) Auxin uses in the control of flowering and fruiting. Annu Rev Plant Physiol 9: 281–310.

[118] Gangappa SN, Chattopadhyay S (2010) MYC2, a bHLH transcription factor, modulates the adult phenotype of SPA1. Plant Signal Behav 5: 1650–1652. 10.4161/psb.5.12.13981 21512327 PMC3115125

[119] Makkena S, Lamb RS (2013) The bHLH transcription factor SPATULA is a key regulator of organ size in *Arabidopsis thaliana* . Plant Signal Behav 8: e24140 10.4161/psb.24140 23470719 PMC3897497

[120] Cifuentes-Esquivel N, Bou-Torrent J, Galstyan A, Galllemi M, Sessa G, et al (2013) The bHLH proteins BEE and BIM positively modulate the shade avoidance syndrome in *Arabidopsis* seedlings. Plant J 75: 989–1002. 10.1111/tpj.12264 23763263

[121] Friedrichsen DM, Nemhauser J, Muramitsu T, Maloof JN, Alonso J, et al (2002) Three redundant brassinosteroid early response genes encode putative bHLH transcription factors required for normal growth. Genetics 162: 1445–1456. 12454087 10.1093/genetics/162.3.1445 PMC1462317

[122] Filiault DL, Maloof JN (2012) A genome-wide association study identifies variants underlying the *Arabidopsis thaliana* shade avoidance response. PLoS Genet 8: e1002589 10.1371/journal.pgen.1002589 22438834 PMC3305432

[123] Kumar R, Ichihashi Y, Kimura S, Chitwood DH, Headland LR, et al (2012) A high-throughput method for Illumina RNA-Seq library preparation. Front Plant Sci 3: 202 10.3389/fpls.2012.00202 22973283 PMC3428589

[124] Nozue K, Covington MF, Duek PD, Lorrain S, Fankhauser C, et al (2007) Rhythmic growth explained by coincidence between internal and external cues. Nature 448: 358–361. 17589502 10.1038/nature05946

[125] Abramoff MD, Magalhaes PJ, Ram SJ (2004) Image processing with ImageJ. Biophotonics International 11: 36–42.

[126] Trapnell C, Pachter L, Salzberg S (2009) TopHat: discovering splice junctions with RNA-Seq. Bioinformatics 25: 1105–1111. 10.1093/bioinformatics/btp120 19289445 PMC2672628

[127] Robinson MD, McCarthy DJ, Smyth GK (2010) edgeR: a Bioconductor package for differential expression analysis of digital gene expression data. Bioinformatics 26: 139–140. 10.1093/bioinformatics/btp616 19910308 PMC2796818

[128] R_Development_Core_Team (2005) R: A language and environment for statistical computing R Foundation for Statistical Computing. Vienna, Austria.

[129] Young MD, Wakefield MJ, Smyth GK, Oshlack A (2010) Gene ontology analysis for RNA-seq: accounting for selection bias. Genome Biol 11: R14 10.1186/gb-2010-11-2-r14 20132535 PMC2872874

[130] Nemhauser JL, Hong F, Chory J (2006) Different plant hormones regulate similar processes through largely nonoverlapping transcriptional responses. Cell 126: 467 16901781 10.1016/j.cell.2006.05.050

[131] Zentella R, Zhang Z-L, Park M, Thomas SG, Endo A, et al (2007) Global analysis of DELLA direct targets in early gibberellin signaling in *Arabidopsis* . Plant Cell 19: 3037–3057. 17933900 10.1105/tpc.107.054999 PMC2174696

[132] Carbon S, Ireland A, Mungall CJ, Shu S, Marshall B, et al (2009) AmiGO: online access to ontology and annotation data. Bioinformatics 25: 288–289. 10.1093/bioinformatics/btn615 19033274 PMC2639003

[133] Bates D, Maechler M, Bolker B, Walker S (2014) lme4: Linear mixed-effects models using Eigen and S4. http://CRAN.R-project.org/package=lme4.

[134] Kuznetsova A, Brockhoff PB, Christensen RHB (2014) lmerTest: Tests for random and fixed effects for linear mixed effect models (lmer objects of lme4 package). http://CRAN.R-project.org/package=lmerTest.

[135] Benjamini Y, Hochberg Y (1995) Controlling the false discovery rate—a practical and powerful approach to multiple testing. J Roy Statist Soc Ser B 57: 289–300.

[136] Winter D, Vinegar B, Nahal H, Ammar R, Wilson GV, et al (2007) An "Electronic Fluorescent Pictograph Browser" for exploring and analyzing large-scale biological data sets. PLoS ONE 2: e718 17684564 10.1371/journal.pone.0000718 PMC1934936

