# Supplementary figures and images for: Shade Avoidance Components and Pathways in Adult Plants Revealed by Phenotypic Profiling
Source: PLoS Genet. 2015 Apr 15;11(4):e1004953. doi: 10.1371/journal.pgen.1004953 (PMC4398415; doi:10.1371/journal.pgen.1004953)

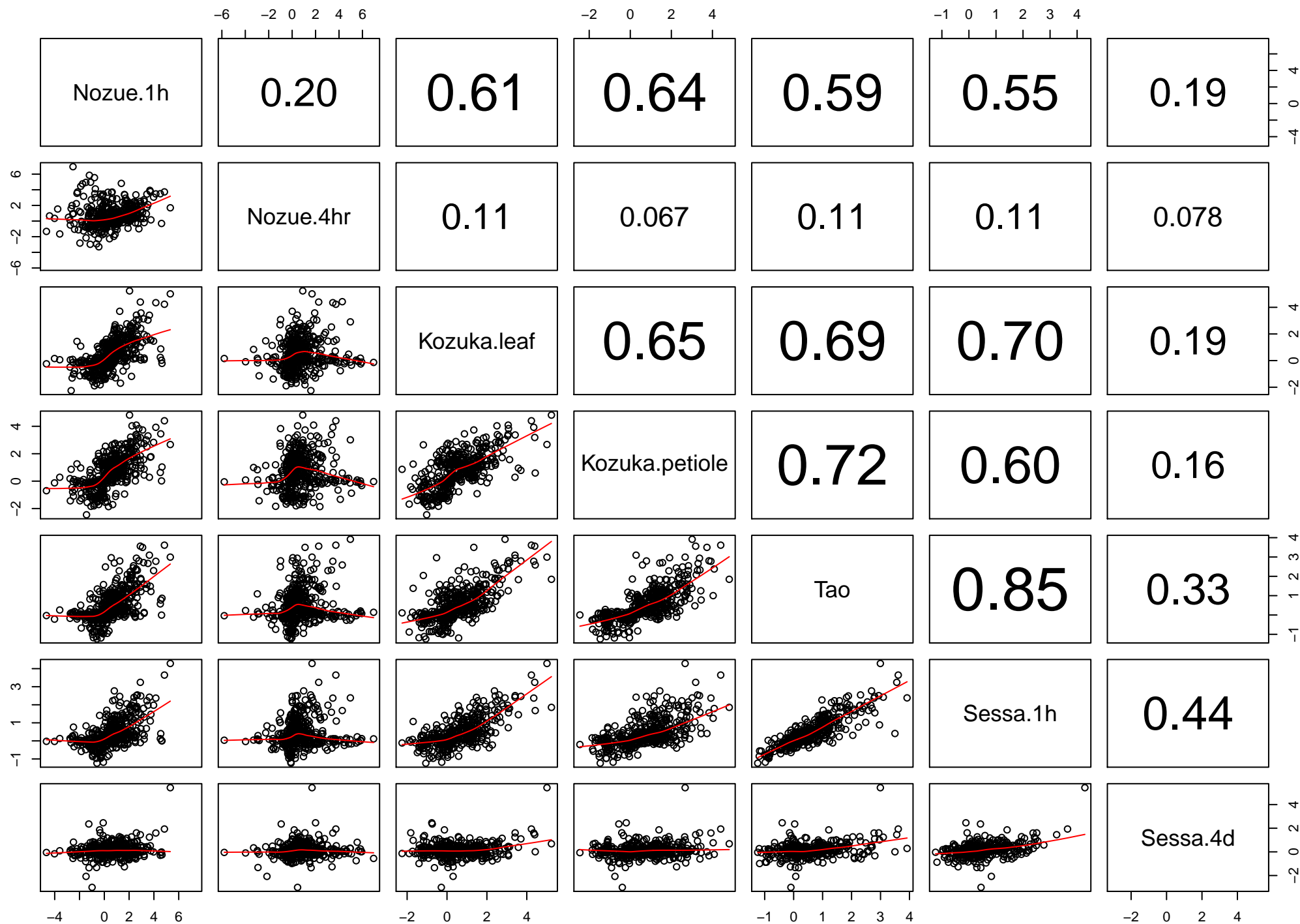

Supplement: S1 Fig — Current RNA-seq data (juvenile plants under low R:FR labeled as “Nozue”) and microarray (leaf or petiole treated with EODFR [6] labeled as “Kozuka”), and hypocotyl treated with low R:FR (1 hour [7,26] (labeled as “Tao” and “Sessa.1h”, 4 days [26] labeled as “Sessa.4d”)). (PDF) [file pgen.1004953.s001.pdf]

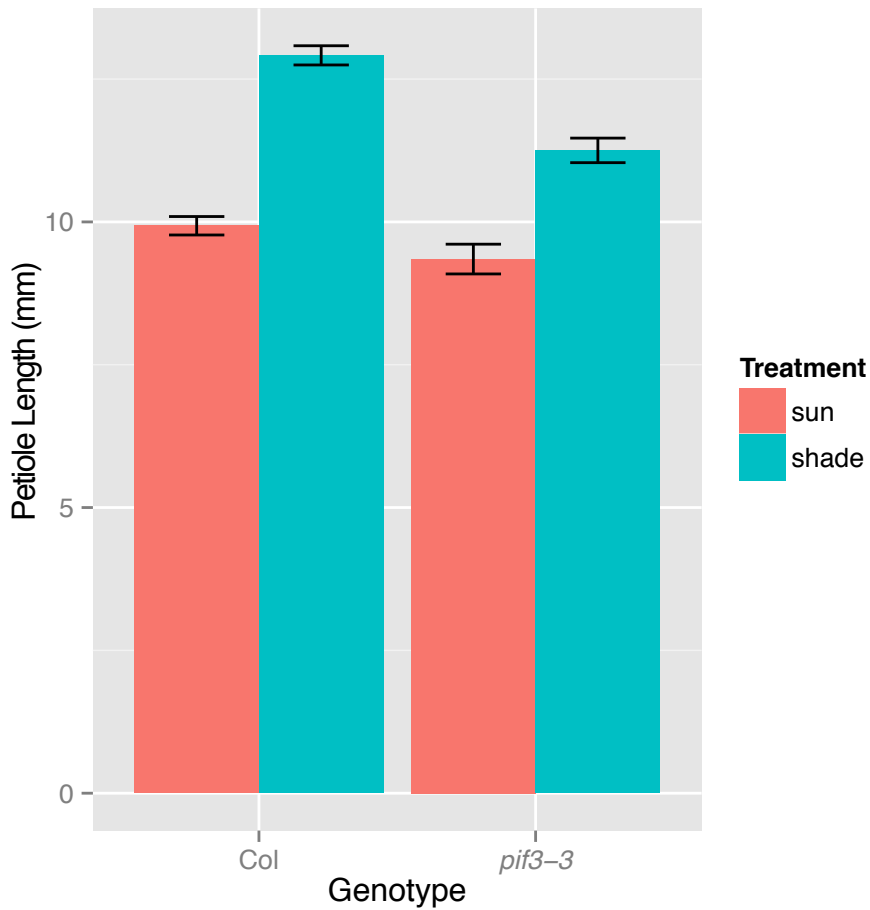

Supplement: S4 Fig — Plants were grown in simulated sun and shade using our standard conditions. Three independent experiments were performed and a total of 20 to 41 plants were examined per treatment/genotype combination. pif3-3 has a significantly reduced response to shade (p<0.01 for genotype X treatment interaction in linear regression). (PDF) [file pgen.1004953.s004.pdf]

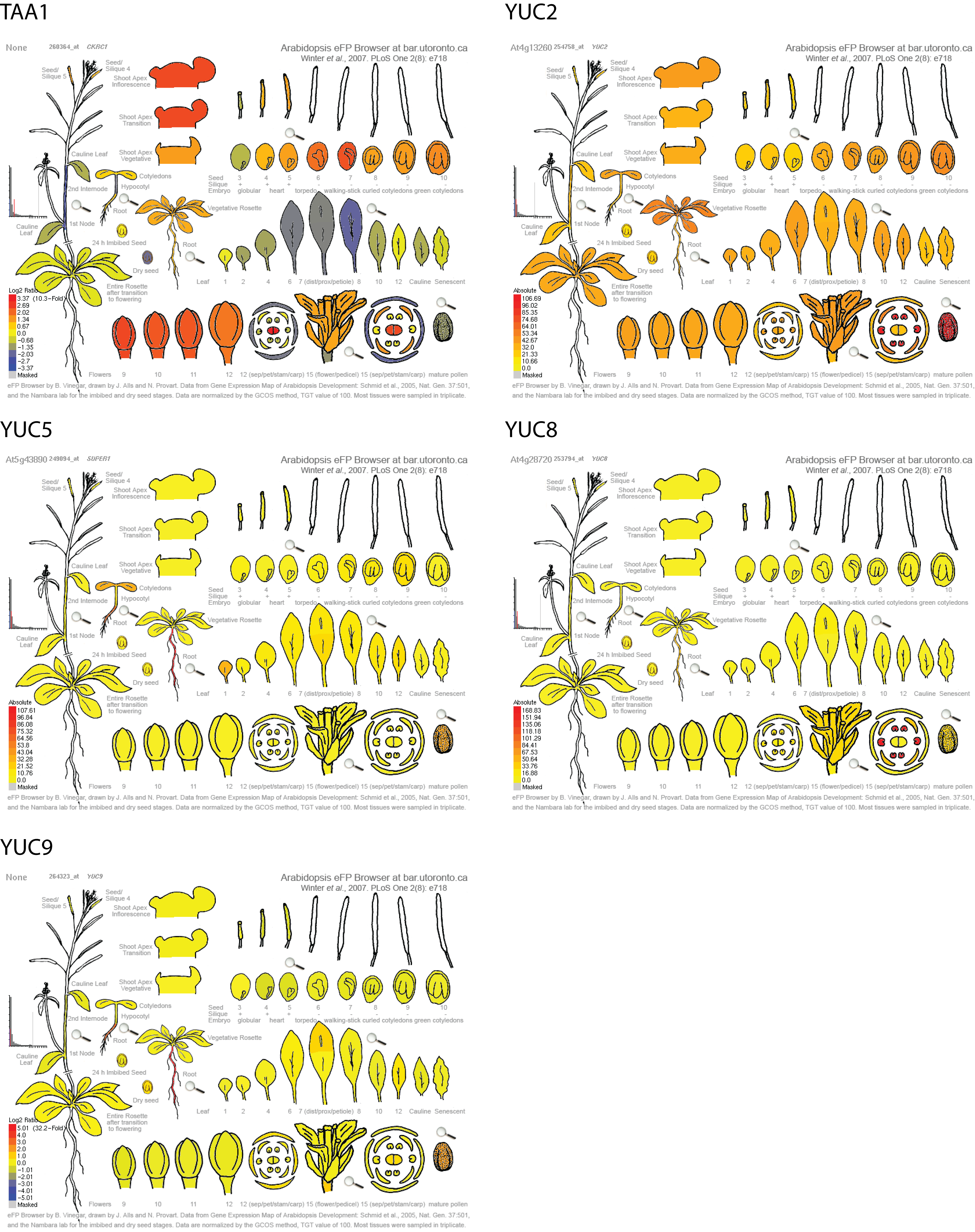

Supplement: S5 Fig — Developmental expression pattern was obtained from eFP browser [136]. (TIF) [file pgen.1004953.s005.tif]

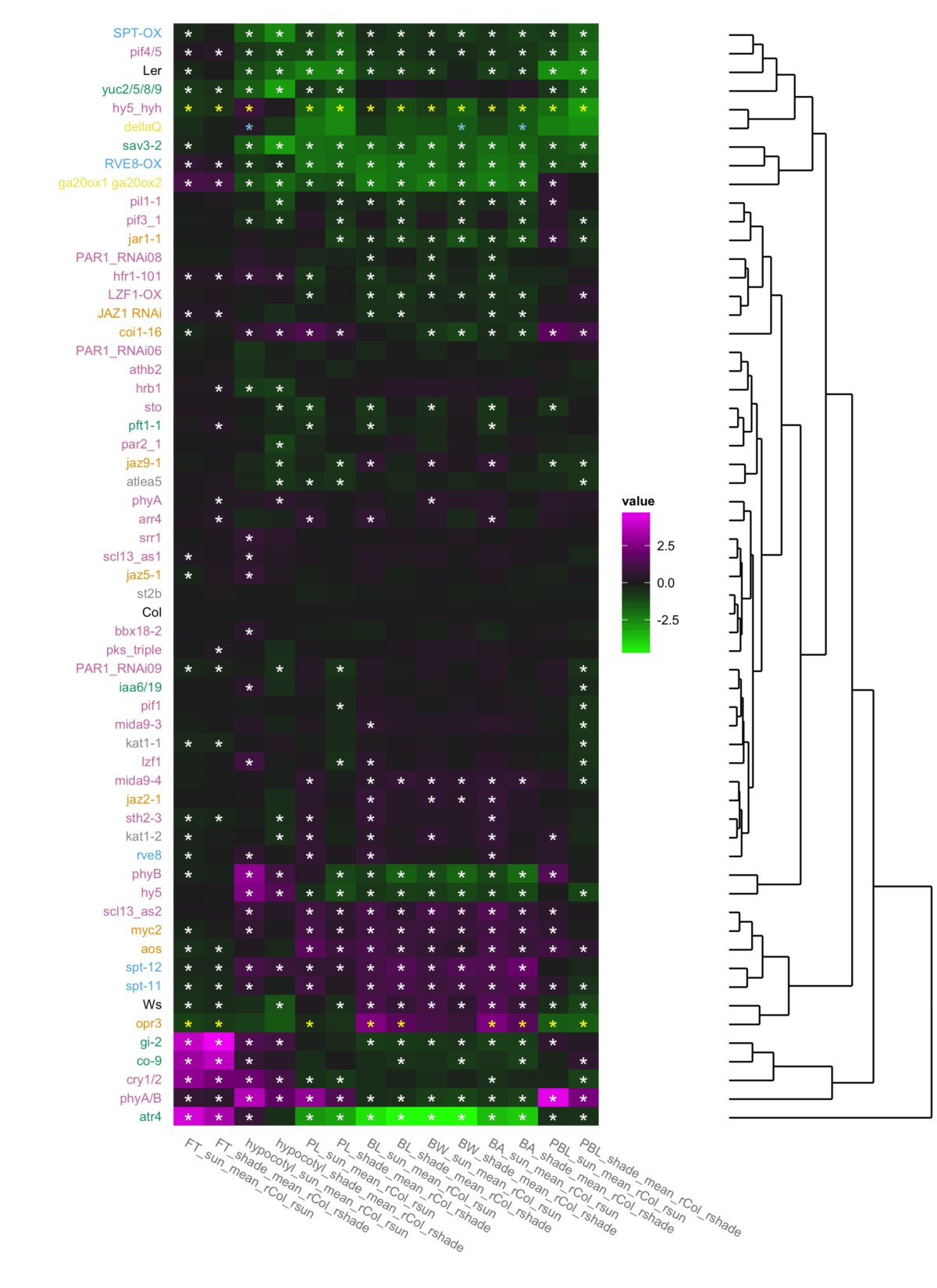

Supplement: S6 Fig — Values were normalized and centered on Col (i.e., Col value = 0) and visualized with color coding (magenta indicates larger value than Col while green indicates smaller value relative to Col). Colors of asterisks indicate genetic background of each mutant, i.e., Col (white), Ws (yellow), and Ler (light blue). (TIF) [file pgen.1004953.s006.tif]
