# Supplementary material for: Shade Avoidance Components and Pathways in Adult Plants Revealed by Phenotypic Profiling
Source: PLoS Genet. 2015 Apr 15;11(4):e1004953. doi: 10.1371/journal.pgen.1004953 (PMC4398415; doi:10.1371/journal.pgen.1004953)

Figure S2A hypocotyl

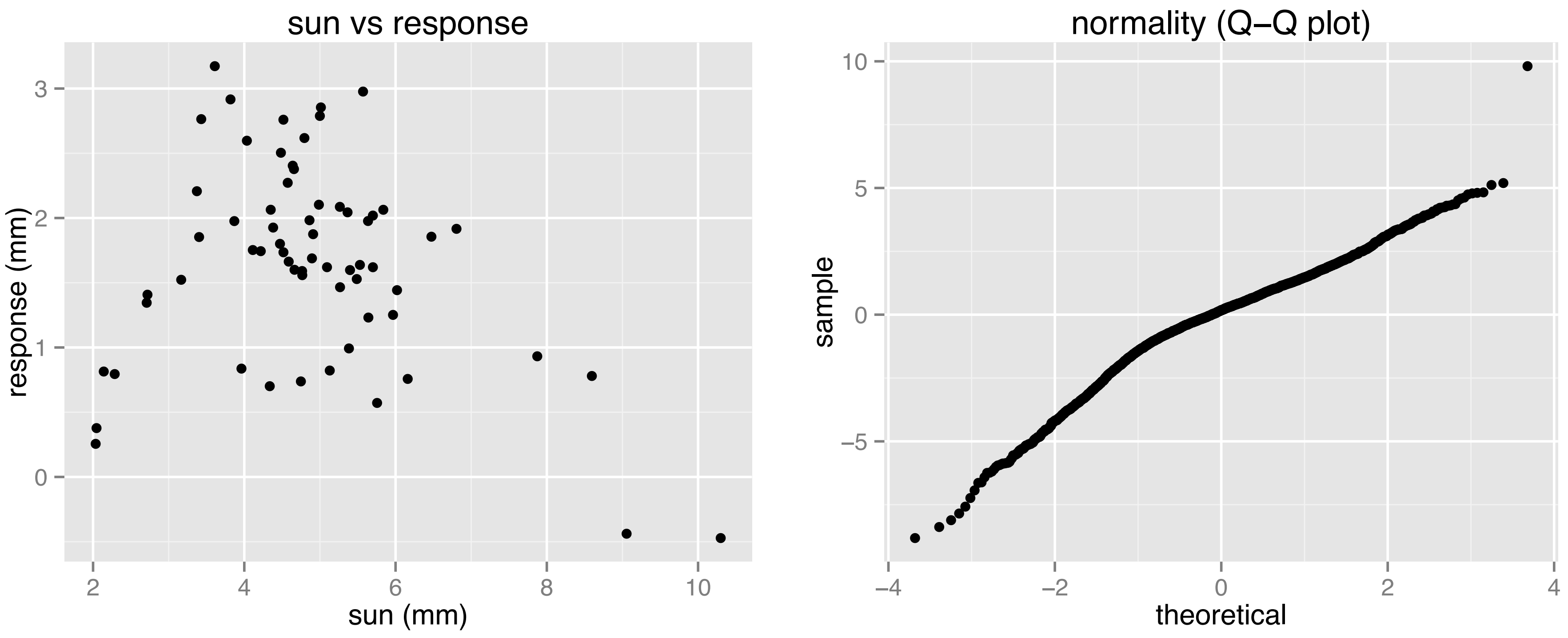

Figure S2B. PL

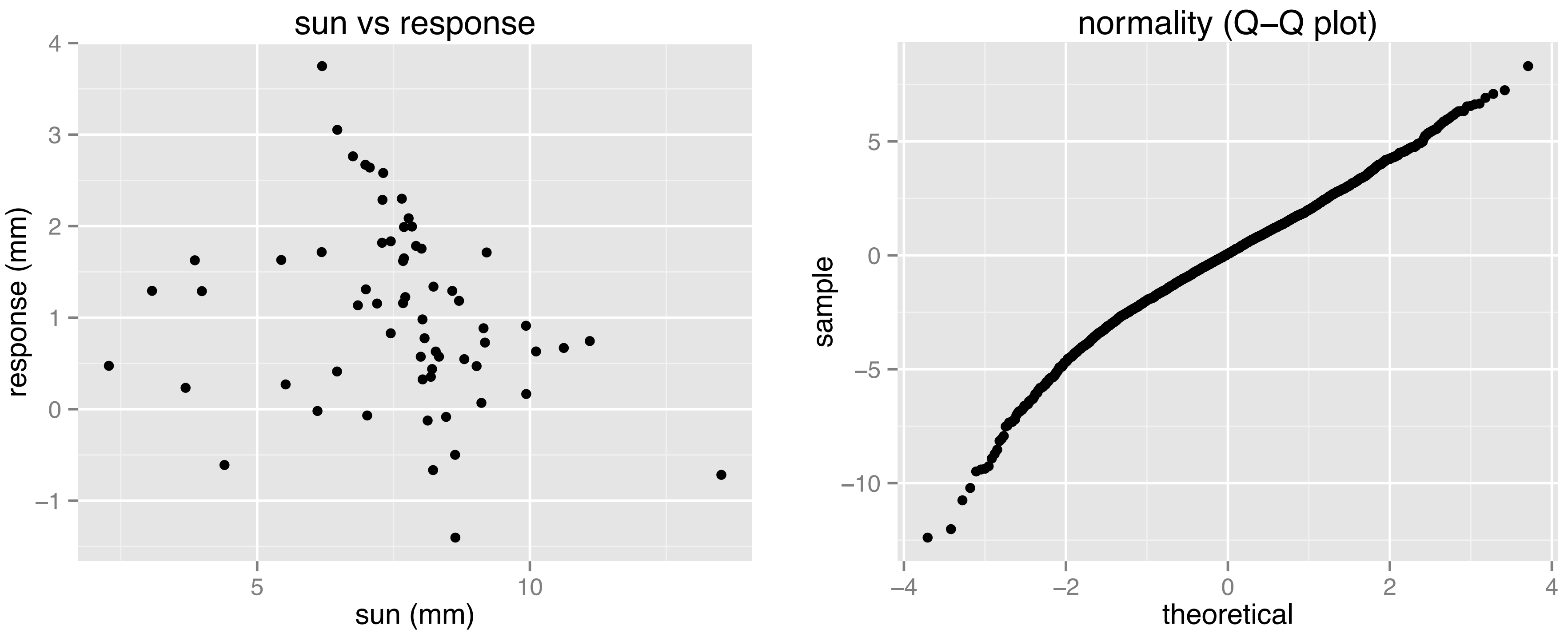

Figure S2C. BL

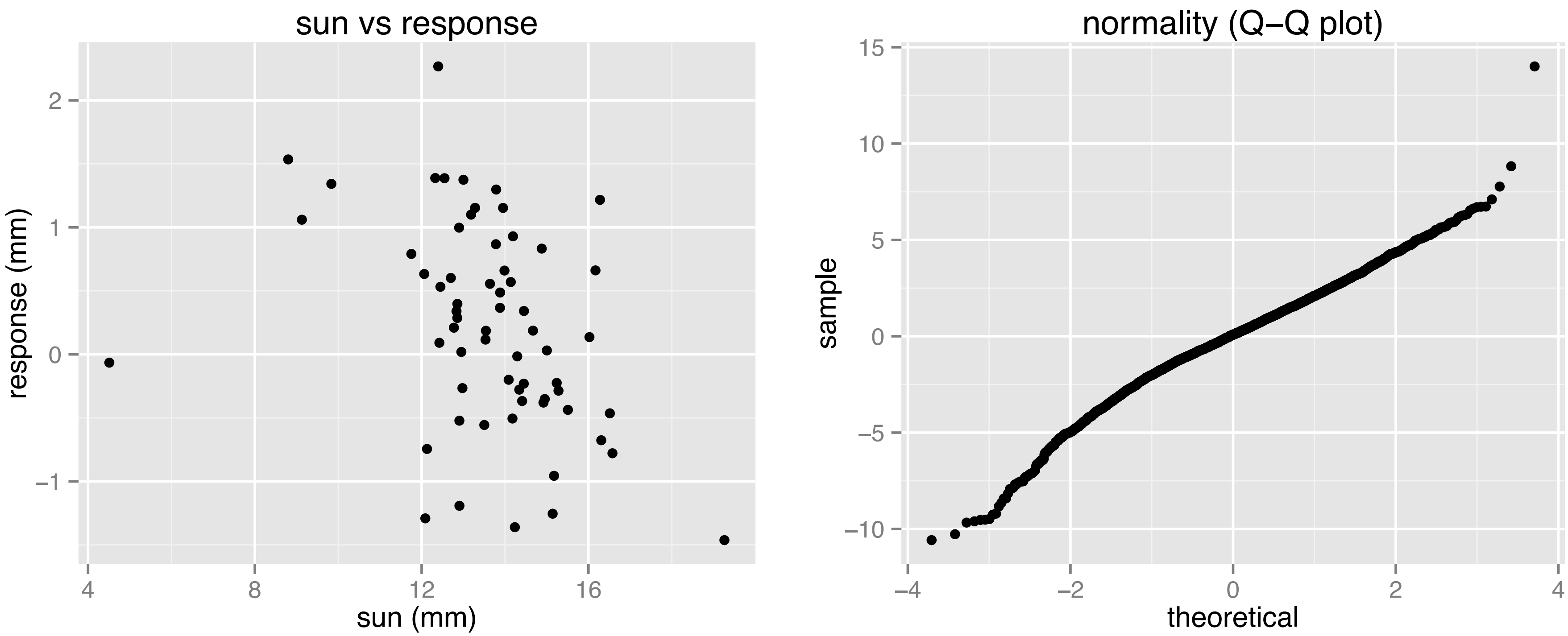

Figure S2D. BW

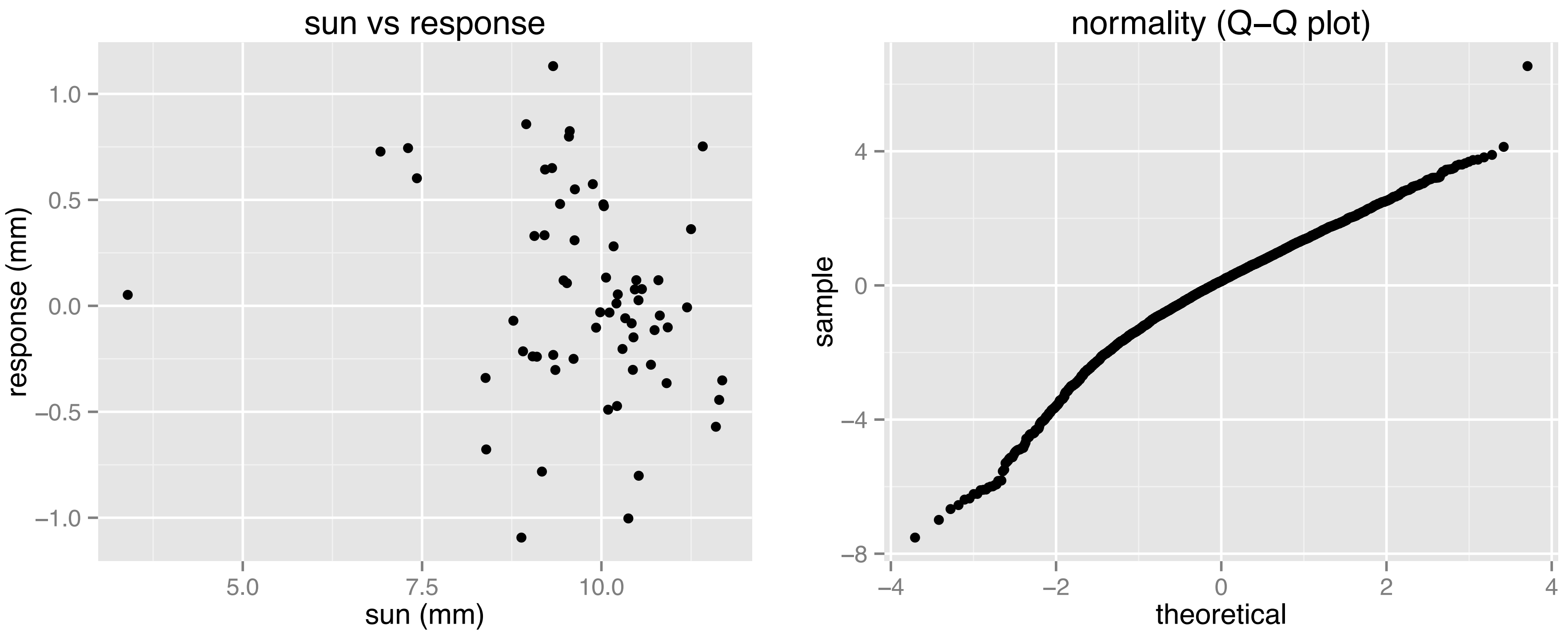

Figure S2E. BA

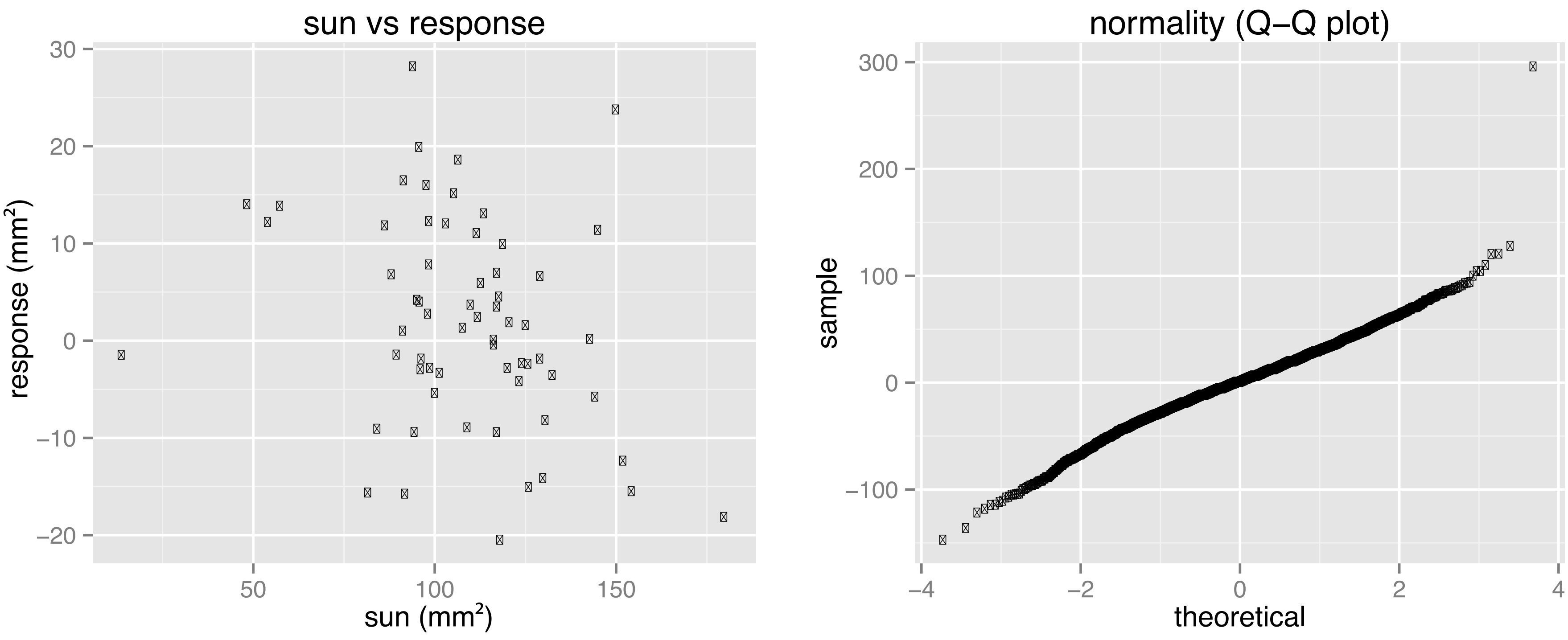

Figure S2F. PBL

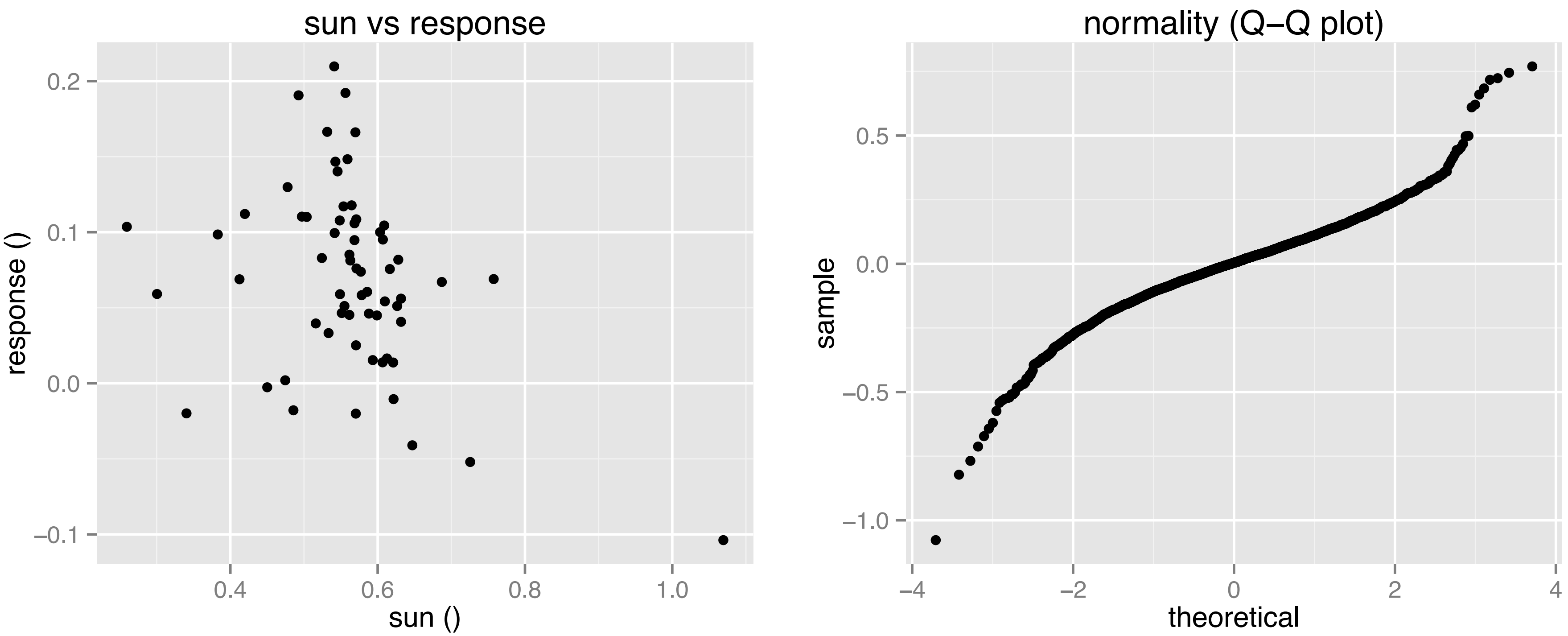

Figure S2G. FT

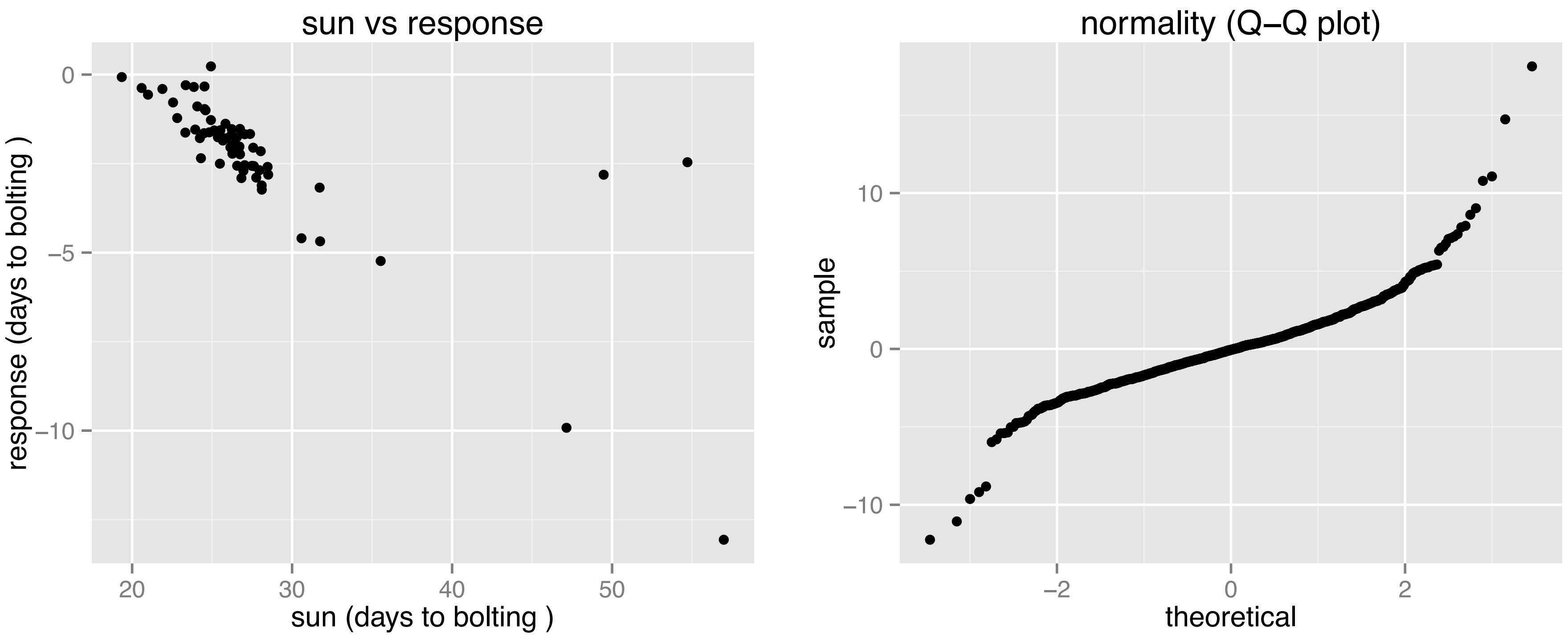

Figure S2H. log2FT

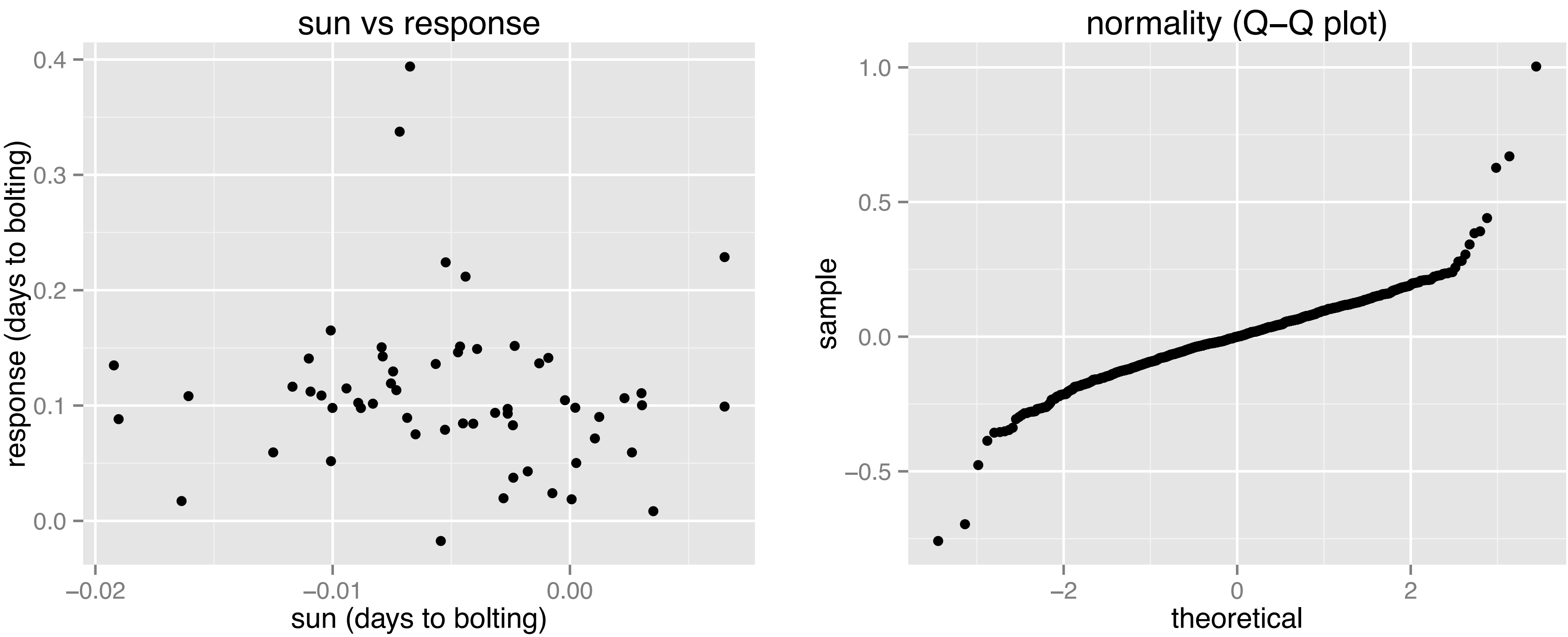

Supplement: S2 Fig — (A) hypocotyl, (B) petiole length, (C) leaf blade length, (D) leaf blade width, (E) leaf blade area, (F) petiole length/leaf blade length ratio, (G) flowering time, and (H) flowering time (log2 transformed). (PDF) [file pgen.1004953.s002.pdf]
