# Supplementary material for: Shade Avoidance Components and Pathways in Adult Plants Revealed by Phenotypic Profiling
Source: PLoS Genet. 2015 Apr 15;11(4):e1004953. doi: 10.1371/journal.pgen.1004953 (PMC4398415; doi:10.1371/journal.pgen.1004953)
Supplement: S3 Fig — (A) hypocotyl, (B) petiole length, (C) leaf blade length, (D) leaf blade width, (E) leaf blade area, (F) petiole length/leaf blade length ratio, (G) flowering time (log2 transformed) and (H) flowering time (log2 transformed residuals). Error bars in (A) to (G) represent standard errors. Genotype names in (H) indicate lines whose flowering time shade response differs significantly from prediction by regression (p < 0.05). (PDF) [file pgen.1004953.s003.pdf]

Figure S3A. hypocotyl

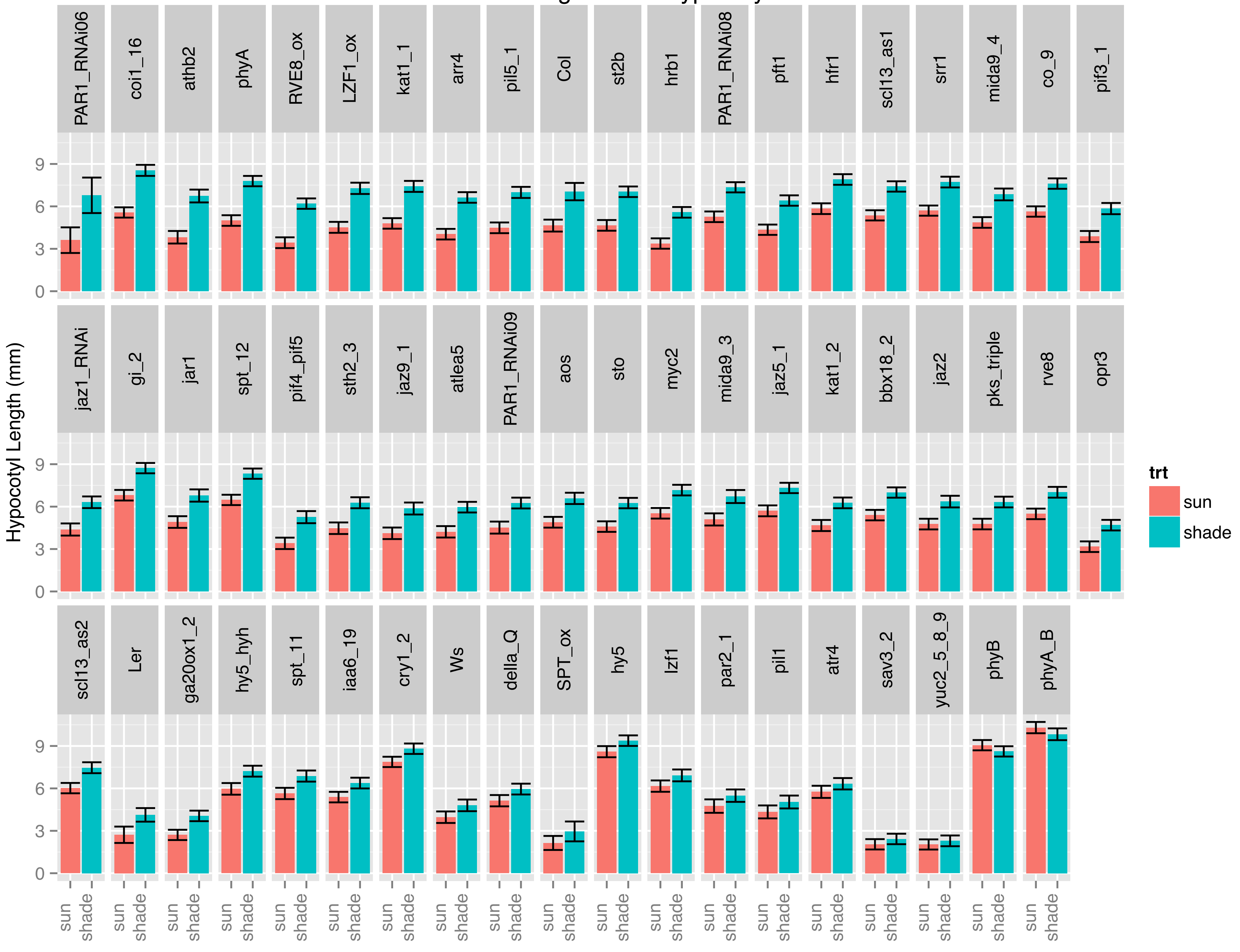

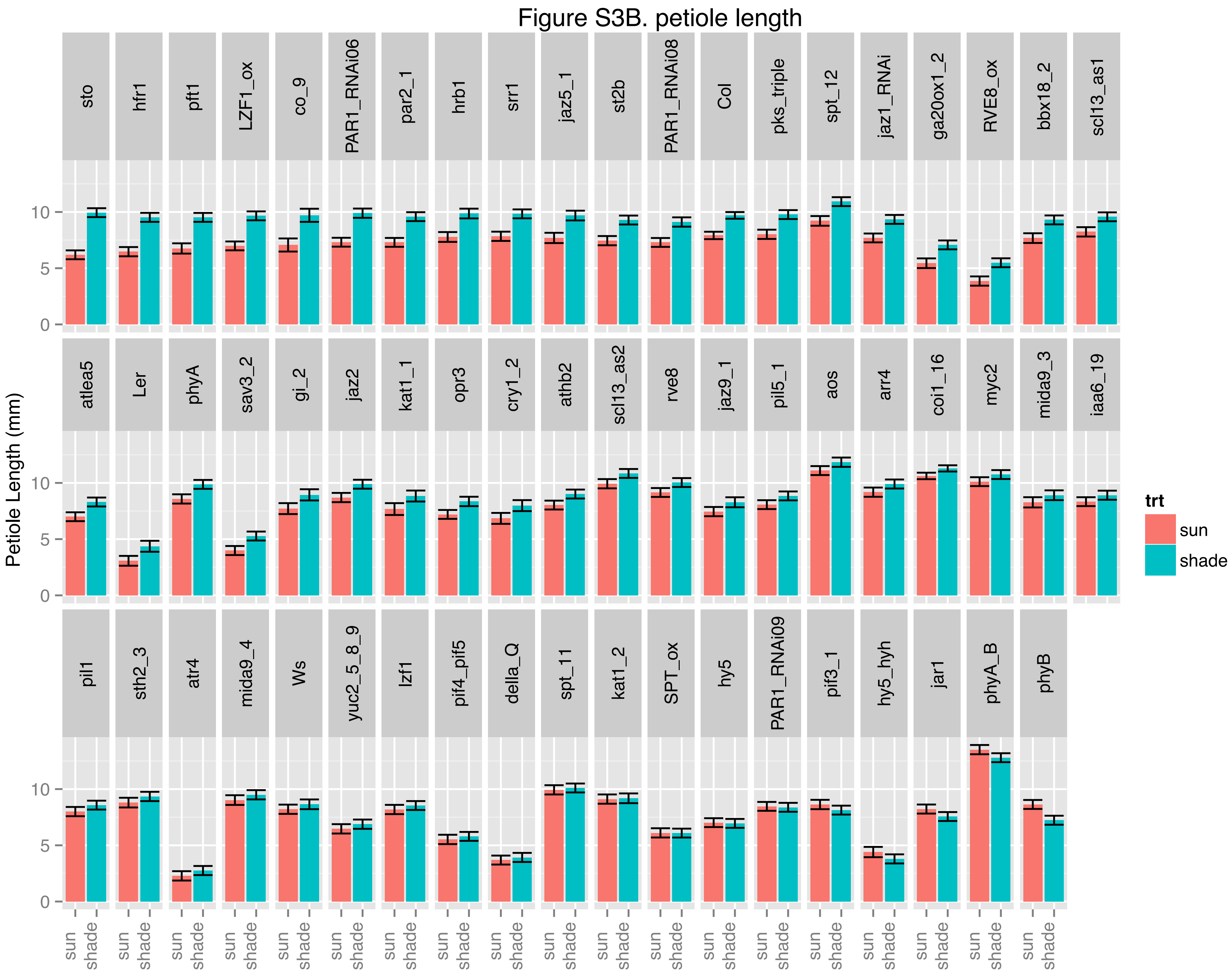

Figure S3B. petiole length
sto
hfr1
pft1
LZF1_ox
co_9
PAR1_RNAi06
par2_1
hrb1
srr1
jaz5_1
st2b
PAR1_RNAi08
Col
pks_triple
spt_12
jaz1_RNAi
ga20ox1_2
RVE8_ox
bbx18_2
scl13_as1
atlea5
Ler
phyA
sav3_2
gi_2
jaz2
kat1_1
opr3
cry1_2
athb2
scl13_as2
rve8
jaz9_1
pil5_1
aos
arr4
coi1_16
myc2
mida9_3
iaa6_19
pil1
sth2_3
atr4
mida9_4
Ws
yuc2_5_8_9
lzf1
pif4_pif5
della_Q
spt_11
kat1_2
SPT_ox
hy5
PAR1_RNAi09
pif3_1
hy5_hyh
jar1
phyA_B
phyB
Petiole Length (mm)
0
5
10
sun
shade
trt
sun
shade

Figure S3C. blade length

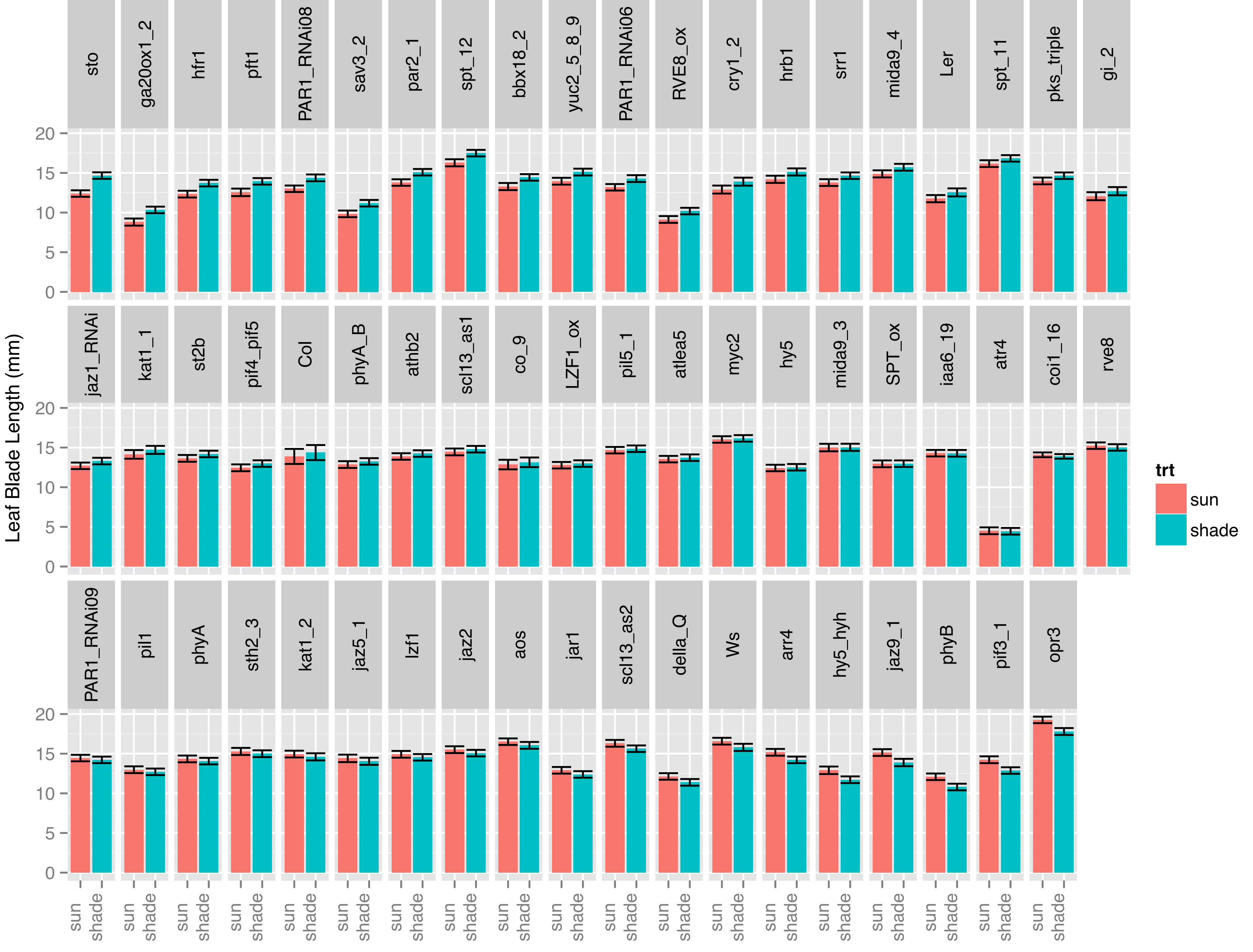

Figure S3D blade width

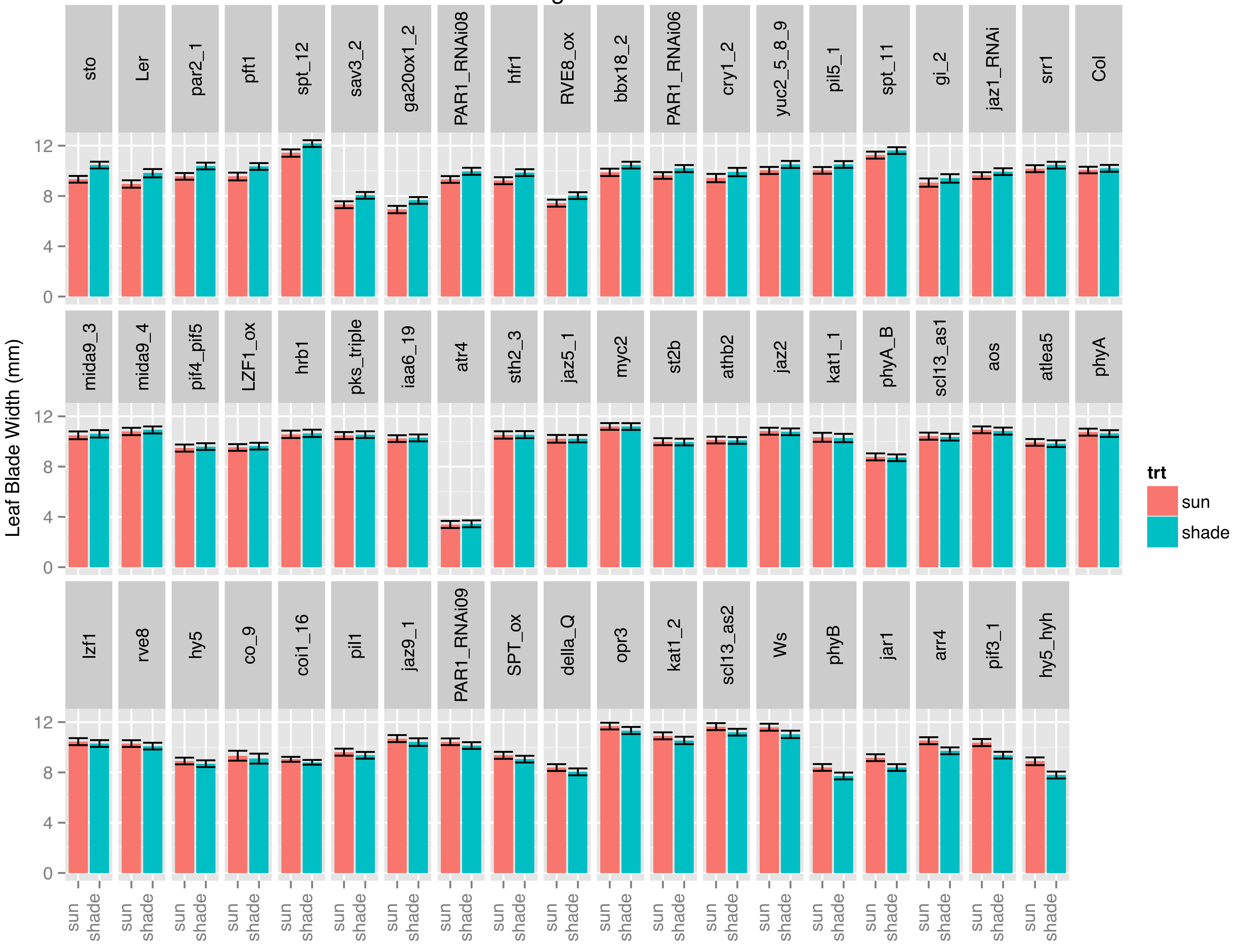

Figure S3E. blade area

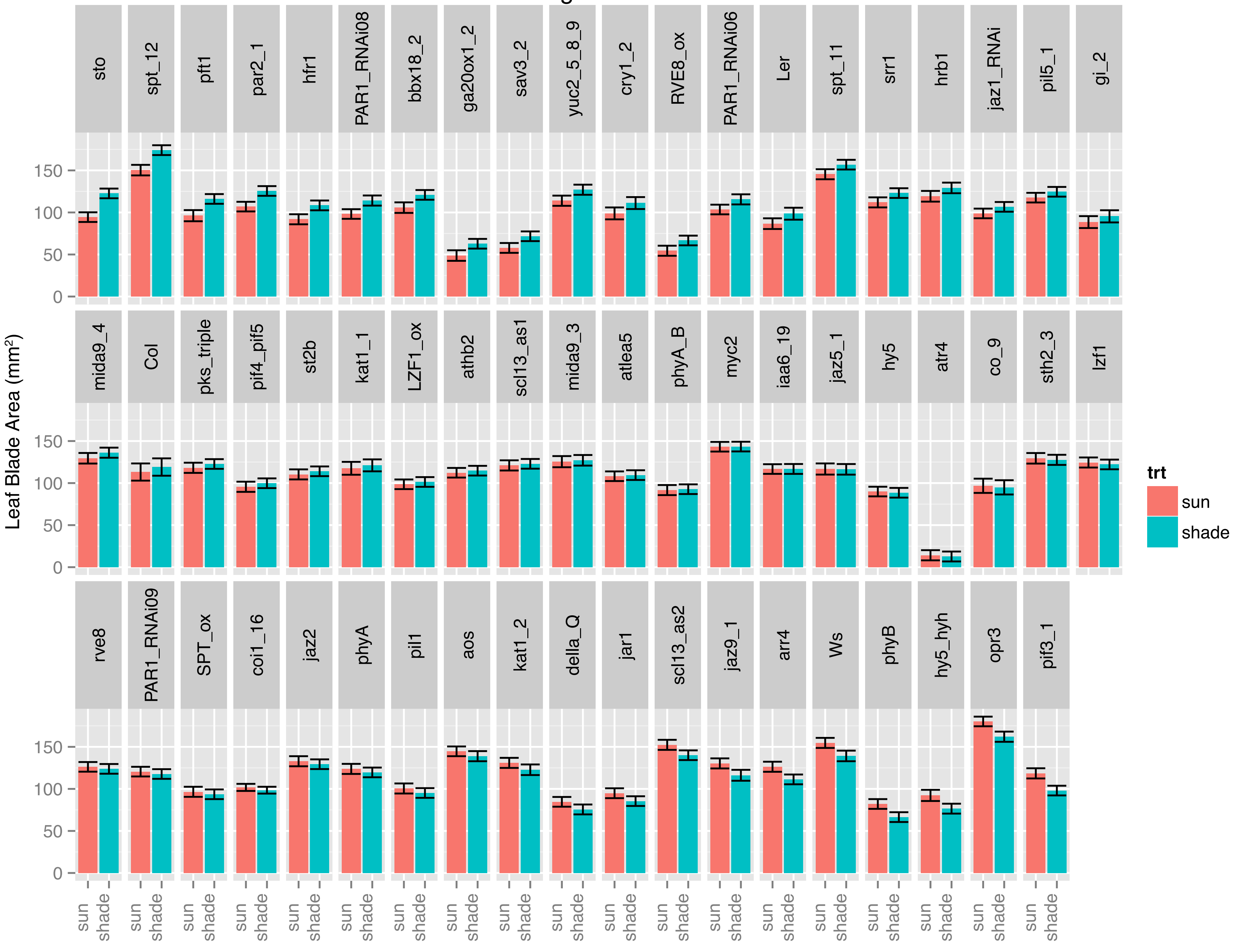

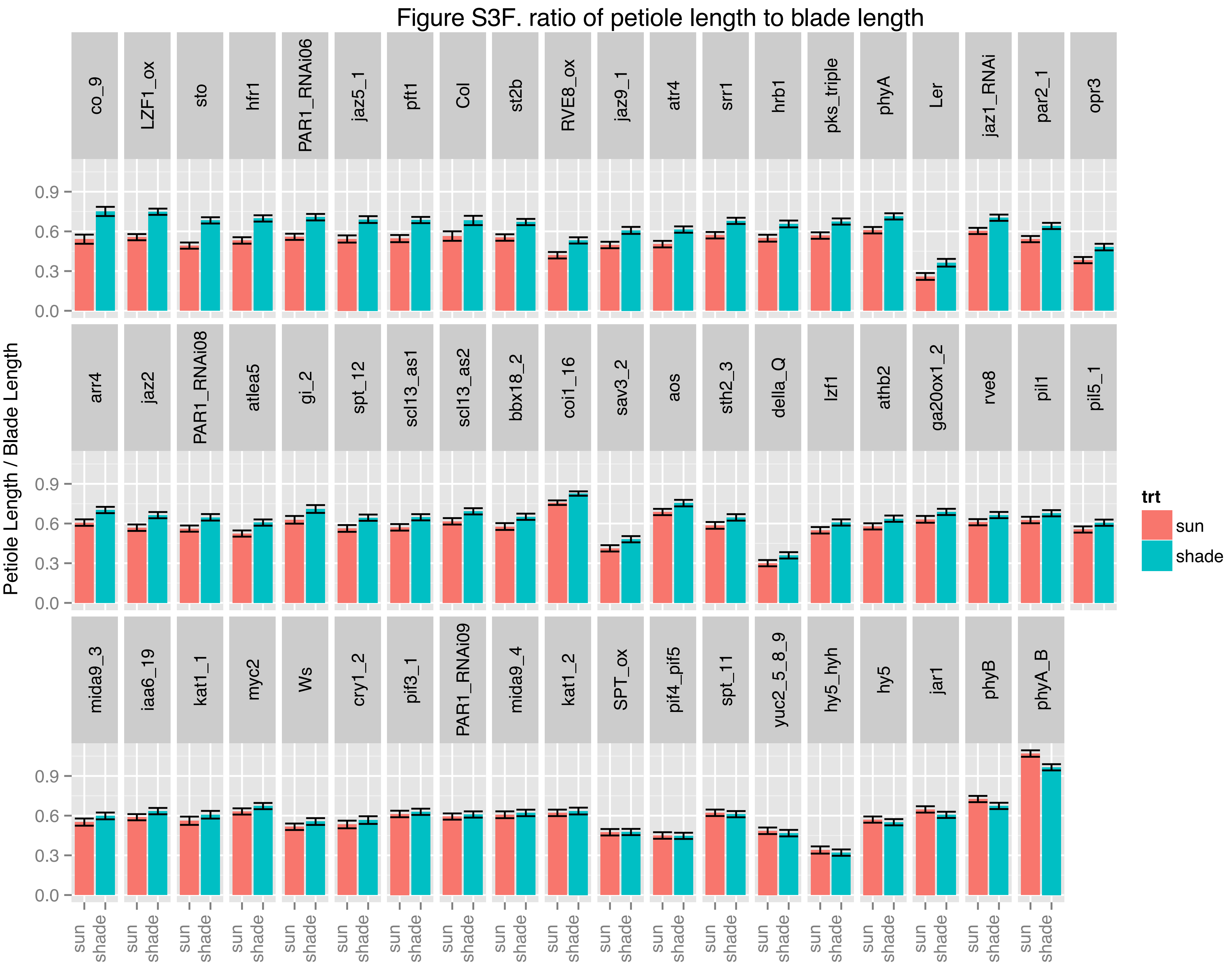
Figure S3F. ratio of petiole length to blade length
Petiole Length / Blade Length
0.0
0.3
0.6
0.9
co_9
LZF1_ox
sto
hfr1
PAR1_RNAi06
jaz5_1
pft1
Col
st2b
RVE8_ox
jaz9_1
atr4
srr1
hrb1
pks_triple
phyA
Ler
jaz1_RNAi
par2_1
opr3
arr4
jaz2
PAR1_RNAi08
atlea5
gi_2
spt_12
scl13_as1
scl13_as2
bbx18_2
coi1_16
sav3_2
aos
sth2_3
della_Q
lzf1
athb2
ga20ox1_2
rve8
pil1
pil5_1
mida9_3
iaa6_19
kat1_1
myc2
Ws
cry1_2
pif3_1
PAR1_RNAi09
mida9_4
kat1_2
SPT_ox
pif4_pif5
spt_11
yuc2_5_8_9
hy5_hyh
hy5
jar1
phyB
phyA_B
sun
shade
trt
sun
shade

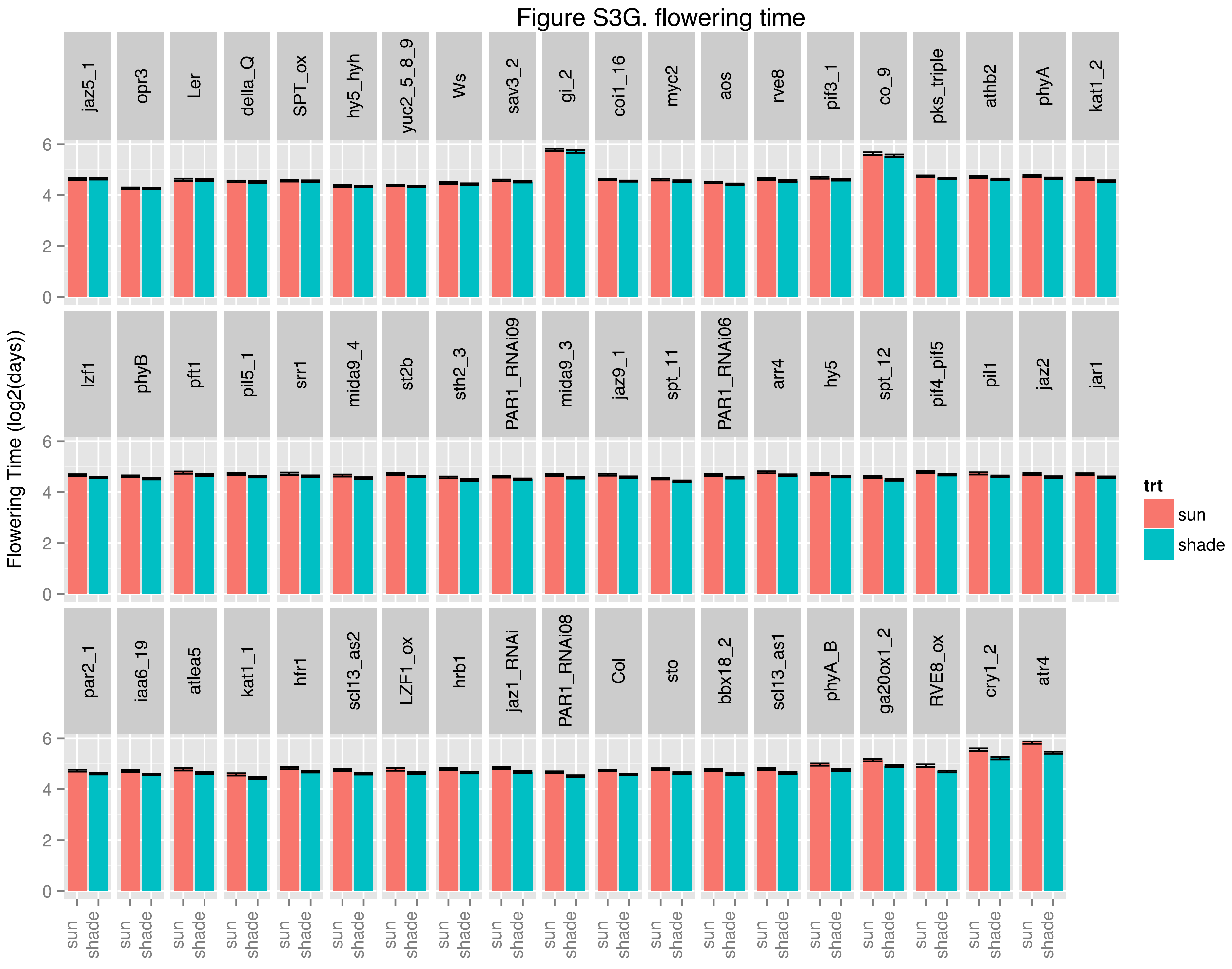

Figure S3G. flowering time
Flowering Time (log2(days))
jaz5_1
opr3
Ler
della_Q
SPT_ox
hy5_hyh
yuc2_5_8_9
Ws
sav3_2
gi_2
coi1_16
myc2
aos
rve8
pif3_1
co_9
pks_triple
athb2
phyA
kat1_2
lzf1
phyB
pft1
pil5_1
srr1
mida9_4
st2b
sth2_3
PAR1_RNAi09
mida9_3
jaz9_1
spt_11
PAR1_RNAi06
arr4
hy5
spt_12
pif4_pif5
pil1
jaz2
jar1
par2_1
iaa6_19
atlea5
kat1_1
hfr1
scl13_as2
LZF1_ox
hrb1
jaz1_RNAi
PAR1_RNAi08
Col
sto
bbx18_2
scl13_as1
phyA_B
ga20ox1_2
RVE8_ox
cry1_2
atr4
0
2
4
6
sun
shade
trt
sun
shade

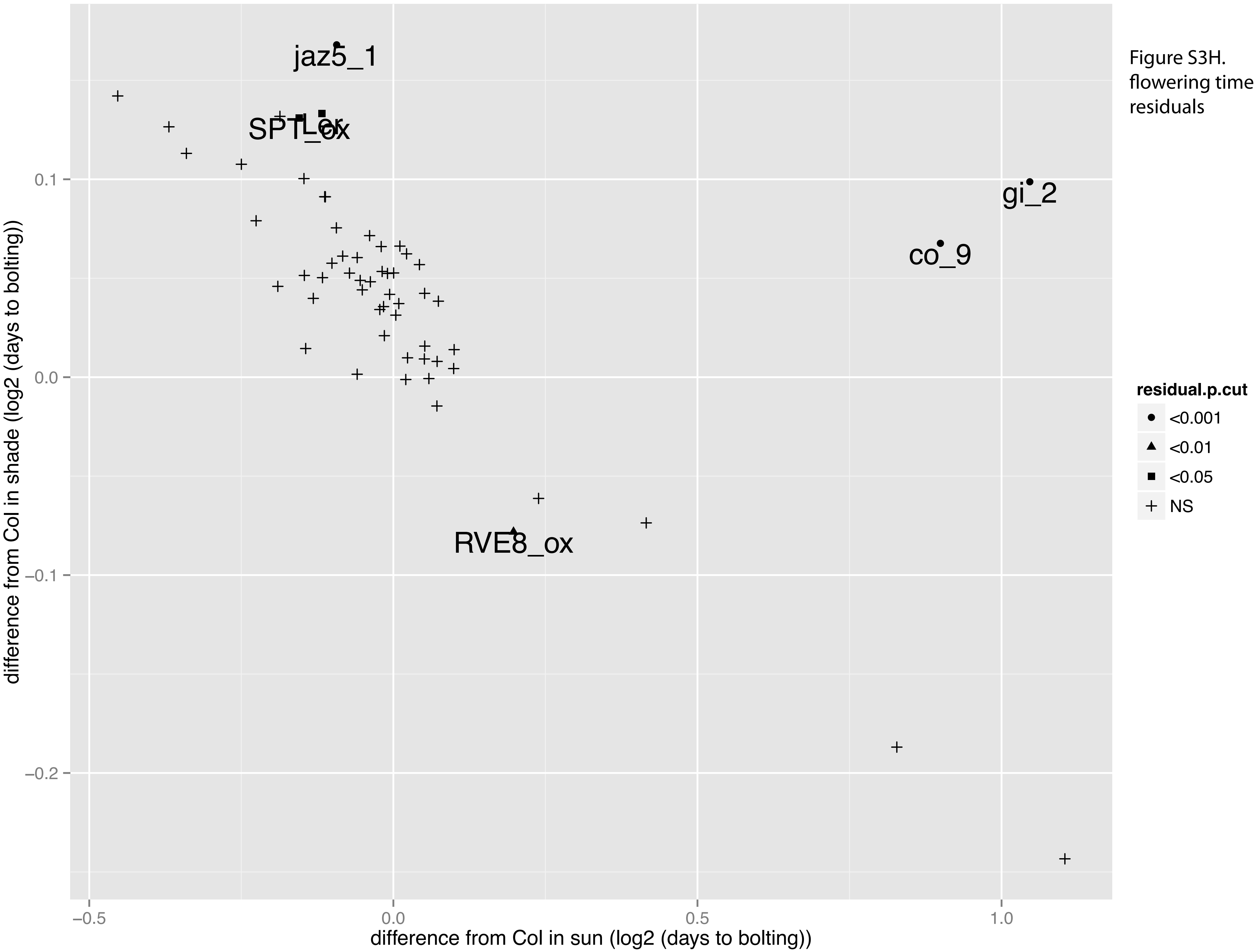

Figure S3H.
flowering time
residuals
